# Comparisons of phenolic components and anti-metabolic activities during different cultivars and growing conditions of blueberry leaves

**DOI:** 10.1016/j.crfs.2025.101256

**Published:** 2025-11-24

**Authors:** Linhang Han, Shuai Sun, Yuqi Yang, Yiling Chen, Gangqiang Dong, Tingzhao Li, Yiming Li, Liuqiang Zhang

**Affiliations:** a School of Pharmacy, Shanghai University of Traditional Chinese Medicine, Shanghai, 201203, China; b Amway (Shanghai) Innovation & Science Co., Ltd., Shanghai, 201203, China; c Amway (China) Botanical R&D Center, Wuxi, 214115, China

**Keywords:** Blueberry leaves, Phenolic compounds, Metabolic regulation, Component-efficacy correlation analysis

## Abstract

Blueberry leaves (BBL), the primary byproduct of blueberry processing industry, are rich in polyphenolic compounds with significant nutritional and medicinal potential. This study established and optimized analytical methods to determine the major components and bioactivities in 110 BBL samples. The results indicated that chlorogenic acid (7.40–102.26 mg/g) was the predominant monomeric component in BBL, with TPC (245.22 ± 67.47 mg/g) generally exceeding TFC (19.61 ± 13.92 mg/g). In terms of cultivar varieties, rabbiteye BBL demonstrated the highest values in neochlorogenic acid (3.60 ± 1.28 mg/g), isoquercitrin (5.06 ± 2.88 mg/g), total phenolics (315.19 ± 52.74 mg/g), antioxidant activities (DPPH: 627.07 ± 150.25 μmol/g; ABTS: 654.90 ± 98.79 μmol/g), as well as α-glucosidase (90.33 % ± 8.64 %) and pancreatic lipase (53.77 % ± 8.64 %) inhibitory capacities. Southern highbush BBL exhibited higher hyperoside content (3.47 ± 3.10 mg/g) and xanthine oxidase inhibitory activity (62.01 % ± 8.97 %), while northern highbush BBL were characterized by elevated levels of chlorogenic acid (52.89 ± 19.24 mg/g), rutin (2.22 ± 1.01 mg/g), and TFC (20.31 ± 12.54 mg/g). The statistical analysis results revealed significant positive correlations between antioxidant activity and the contents of neochlorogenic acid, chlorogenic acid, total phenolics, and total flavonoids. Specifically, a positive correlation was observed between α-glucosidase inhibition and the contents of total phenolics and flavonoids, while a negative correlation existed between pancreatic lipase inhibition and hyperoside content. The entropy weight method confirmed rabbiteye BBL exhibited superior quality compared to highbush types, and open-field BBL significantly surpassed greenhouse-grown samples. This study investigated the characteristic chemical components and metabolism-related bioactivities of BBL across different cultivars and growing environments. By integrating chemometric analysis for correlation assessment, it provides a scientific basis for the resource utilization and high-value exploitation of BBL.

## Highlights

• Optimized quantitative methods for TPC, TFC, and key phenolics in BBL.
• The inhibitory capacity of BBL samples against three metabolic enzymes was measured.
• Comprehensive evaluation of BBL quality was achieved through entropy weight method.
• Open-field BBL showed higher active component levels than greenhouse-cultivated.
• The quality of rabbiteye BBL surpasses that of both southern and northern highbush.

## Graphical abstract

**Figure:**
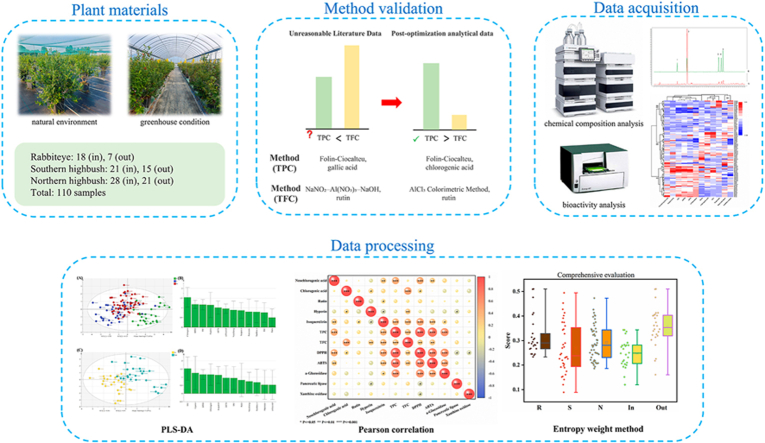

## 1 Introduction

Blueberries (*Vaccinium* spp.), deciduous shrubs within the *Ericaceae* family, are globally acclaimed as the “King of Berries” owing to their distinctive sweet-tart flavor, high nutritional density, diverse array of bioactive constituents, and considerable economic importance. Cultivation of blueberries is widespread across numerous global regions, with primary production centers strategically distributed throughout the Americas, Asia, and Europe. According to the *Global Blueberry Industry Status Report (2024)* from the International Blueberry Organization (IBO, https://www.internationalblueberry.org/), the worldwide blueberry cultivation area reached 262,417 ha, yielding a total production of 1782.26 thousand metric tons (KMT) by the end of 2023. China ranked as the top global producer with 84,420 ha under cultivation and an output of 563.46 KMT. As a major byproduct of blueberry processing, blueberry leaves (BBL) are increasingly recognized for their potential application. This growing interest is fueled not only by the continued expansion of commercial cultivation but also by mounting evidence their beneficial health properties, particularly their abundance of diverse phenolic compounds. Although the study has demonstrated that fermented BBL tea has the potential to improve daily sleep quality and enhance overall quality of life (Shoji et al., 2025), these findings still provide limited direct impetus for the systematic development and industrial transformation of BBL resources.

Native American ethnobotanical records, including seminal works such as the *Handbook of Medicinal Herbs* (Duke, 2002) and *Medicinal Plants of Native America* (Moerman, 1986), explicitly documented the medicinal use of BBL. These leaves were typically prepared as teas or decoctions to support the management of diabetes, digestive disorders, and wound healing. These applications highlight the multifaceted value of BBL, and help to bridge historical ethnobotanical practices and modern validation through herbalism and pharmacological research. Although existing pharmacological research has demonstrated that of BBL extracts mainly exhibited antioxidative (Wu et al., 2019), hypoglycemic (Takács et al., 2020; Hu et al., 2024), and lipid-modulating (Yuji et al., 2013) effects, uric acid-lowering effects (Yamasaki et al., 2021), the most studies to date have only focused on the correlation analysis between polyphenolic compounds in BBL and their antioxidant activity (Qi et al., 2024; Wu et al., 2019). Importantly, the biological activities of polyphenolic compounds in BBL related to their traditional applications in metabolic disorders remain largely unexplored.

BBL represent a rich source of polyphenolic compounds, primarily including phenolic acids, flavonoids, and anthocyanins (Jianbo et al., 2022; Shaoyi et al., 2022). The phenolic acids are largely characterized by caffeoylquinic acid derivatives, including chlorogenic acid, crypto chlorogenic acid, neochlorogenic acid, 3,5-*O*-dicaffeoylquinic acid, and 4,5-*O*-dicaffeoylquinic acid (Wang et al., 2015). The flavonoid profiling reveals a distinct profile characterized by three major flavonols: quercetin, kaempferol, and myricetin, which occur predominantly as glucoside and galactoside derivatives (Wu et al., 2019). The anthocyanin fraction comprises aglycones commonly associated with pigments found in cornflower, delphinium, morning glory, and mallow, along with their corresponding glycosides (Wenhua et al., 2012). However, the non-specificity of the reported methods for quantifying total polyphenols and flavonoids in BBL lead to paradoxical results wherein flavonoid content abnormally exceeds that of total polyphenol (Wu et al., 2019; Wang et al., 2025). Notably, while open-field and greenhouse cultivation represent the primary cultivation environments for blueberries, the correlation between polyphenolic profiles of BBL and their associated bioactivities across these cultivation environments remains unexplored. Furthermore, systematic evaluation methods for phenolic content-bioactivity relationships in BBL are still lacking.

Blueberries encompass numerous varieties, which are mainly classified into northern highbush blueberry (*V*. *corymbosum*), southern highbush blueberry (*V*. *corymbosum*), lowbush blueberry (*V*. *angustifolium*), rabbiteye blueberry (*V*. *virgatum*), and half-high blueberry (*V*. *corymbosum* × *V*. *angustifolium*)(Edger et al., 2022; Xutong et al., 2025). As the dominant cultivar in global commercial production, the northern highbush blueberry has become the preferred choice for large-scale cultivation in temperate to subarctic regions, owing to its exceptional cold hardiness, superior fruit quality, high and stable yields, and broad ecological adaptability (Sinelli et al., 2008). Southern highbush blueberry, characterized by low chilling requirement, strong tolerance to high temperatures and drought, adaptability to poor soils, and strong disease resistance, has become the dominant variety in warm-climate cultivation systems (Nishiyama et al., 2021). Rabbiteye blueberry exhibits strong environment adaptability including drought-tolerance, tolerance to poor soils, resistance to diseases and pests. It can produce large fruits with high sweetness well-suited to the hot and humid conditions of southern regions, and can achieve high yields through soilless cultivation techniques (Xutong et al., 2025). The BBL samples analyzed in this study (n = 110) were sourced from the Amway (China) Botanical R&D Center in Wuxi, a research institute dedicated to the cultivation and development of medicinal plants, which maintains an extensive repository of plant genetic resources within the industry.

This study aims to: (1) optimize and establish analytical methods for quantifying the main characteristic compounds, total polyphenols, and total flavonoids in BBL based on high-performance liquid chromatography (HPLC)-DAD and UV–visible spectrophotometry, and determine the content of these target constituents across different cultivars and growing environments; (2) evaluate the inhibitory effects of bioactive constituents in BBL from various cultivars and growing environments on α-glucosidase, pancreatic lipase, and xanthine oxidase, alongside conventional evaluations of antioxidant activity; (3) utilize multivariate statistical analysis to explore the correlations between chemical composition and bioactivities of BBL across diverse cultivars and growing environments, and integrating multidimensional indicators through entropy weight method to establish a systematic quality evaluation framework for BBL, thereby supporting the development of functional foods and pharmaceuticals.

## 2 Materials and methods

### 2.1 Plant materials and sample preparation

In August 2022, 110 batches of BBL with uniform maturity and no visible pathological characteristics were systematically collected from Amway (China) Botanical R&D Center (ABRC; 31°29′34″ N, 120°31′27″ E, Wuxi, China). All samples were categorized into three cultivated types: rabbiteye blueberry (*V virgatum*, n = 25), southern highbush blueberry (*V. corymbosum*, n = 36), and northern highbush blueberry (*V. corymbosum*, n = 49). Each cultivated type was further stratified into two subgroups according to growth environments: greenhouse cultivation (designated as ‘indoor’) and open-field cultivation (‘outdoor’). To ensure sample confidentiality while maintaining traceability, an anonymized coding system was applied using “R/S/N/H + Arabic numerals + (in/out)" to reflect cultivar category and growth condition (see Table S1 for details). For each batch, no less than 100 g of mature leaves were collected, and transported to the laboratory as promptly as possible for storage at −20 °C. All samples were freeze-dried for 48 h using a vacuum freeze dryer, then ground into fine powder with a mechanical grinder, and sieved through a 50-mesh stainless steel sieve (equivalent to 300 μm particle size), respectively. The resulting powder was sealed in plastic bags and stored at −20 °C until further analysis.

### 2.2 Chemicals and reagents

All chemicals and reagents used were of analytical reagent grade or higher. Phosphoric acid (≥85 %, PA), acetonitrile, and methanol (HPLC grade) were purchased from Shanghai Titan Scientific Co., Ltd. (China). Ultrapure water was prepared using a Milli-Q Reference system (Merck Millipore, USA). The analytical standards of neochlorogenic acid (Lot.14,332, >98 %), chlorogenic acid (Lot.15,360, >98 %), rutin (Lot.240,129, >98 %), hyperoside (Lot.10,020, >98 %), and isoquercitrin (Lot.9691, >98 %) were sourced from Shanghai Standard Technology Service Co., Ltd. (China). These compounds were used to prepare standard/calibration curves by HPLC. DPPH (2,2-diphenyl-1-picrylhydrazyl), Trolox, and the Total Antioxidant Capacity Assay Kit (ABTS method) were acquired from Shanghai Beyotime Biotechnology Co., Ltd. (China). α-Glucosidase (from *Saccharomyces cerevisiae*) and lipase (from *porcine pancreas*) were procured from Sigma-Aldrich (Merck KGaA, Germany). Xanthine oxidase and xanthine were supplied by Shanghai Macklin Biochemical Technology Co., Ltd. (China). Other chemicals and reagents were of analytical grade.

### 2.3 Preparation of reference substance solutions and sample solutions

Chlorogenic acid standard was accurately weighed and dissolved in deionized water to prepare a 5.00 mg/mL stock solution. Individual stock solutions of neochlorogenic acid, rutin, hyperoside and isoquercitrin were prepared by dissolving each reference standard in 50 % (v/v) ethanol to obtain at concentrations of 0.28, 0.24, 0.15 and 0.36 mg/mL, respectively. For sample preparation, 250 mg of each BBL powder was accurately weighed into a 10 mL volumetric flask and made up to volume with 50 % ethanol. The flask was weighed again before subjecting it to ultrasonic extraction at 750 W for 15 min. After cooling to room temperature, any loss in weight due to solvent evaporation was replenished with additional 50 % ethanol. Each sample was filtered through 0.22 μm nylon membrane filters prior to analysis.

### 2.4 Determination of the total phenolic content (TPC)

TPC was determined using the Folin-Ciocalteu method (Ainsworth and Gillespie, 2007) with slight modifications. Briefly, the sample solution was diluted 100-fold with ultrapure water, and 1.000 mL of the diluted solution was precisely transferred into a 10 mL brown volumetric flask. Subsequently, 0.500 mL of Folin-Ciocalteu reagent (1.0 mol/L) was added, and the mixture was vortexed for 10 s, followed by reaction in the dark condition at room temperature for 6 min. Then, 4.000 mL of 7.5 % (w/v) sodium carbonate solution was added, and the volume was adjusted to 10 mL with ultrapure water. After 30 min of dark incubation, the absorbance was measured at 765.0 nm using a BioTek Synergy H1 multi-mode microplate reader (BioTek Instruments, USA). TPC was quantified using the chlorogenic acid standard calibration curve and expressed as milligrams of chlorogenic acid equivalent (CAE) per gram of dry weight (mg CAE/g DW).

### 2.5 Determination of the total flavonoid content (TFC)

The determination of TFC was carried based on AlCl_3_ colorimetry (Zhang et al., 2024) with appropriate modifications. Briefly, the sample solution was diluted 10-fold with 50 % ethanol, and 1.000 mL of the diluted solution was accurately pipetted into a 10 mL amber volumetric flask. Subsequently, 1.000 mL of 0.1 M AlCl_3_ solution and 2.000 mL of 0.1 M acetate buffer (pH 5.4) were sequentially added. The mixture was made up to 10 mL with 50 % ethanol, vortex-mixed for 10 s, and incubated at 30 °C for 12 min. The absorbance was measured at 415.0 nm using a BioTek Synergy H1 microplate reader (BioTek Instruments, USA). TFC was calculated based on the rutin standard calibration curve and expressed as rutin equivalent (RTE) in milligrams per gram dry weight (mg RTE/g DW).

### 2.6 Qualitative and quantitative analysis of chemical compounds by HPLC

The analysis of main phenolic compounds in BBL was performed according to 1a previous established method (Qi et al., 2024) with appropriate modifications. The filtered sample solutions were analyzed using an Agilent 1260 HPLC system (Agilent Technologies, USA) equipped with a diode-array detector (DAD). Chromatographic separation was conducted using a Shim-pack GIST C_18_-AQ column [250.0 mm × 4.600 mm, 5.000 μm, Shimadzu (Shanghai) Laboratory Equipment Co., Ltd., China] maintained at 35.00 °C. The optimal mobile phase consisted of acetonitrile (A) and 0.1 % PA in water (B) (v/v), delivered at a flow rate of 1.000 mL/min with the following gradient program: 0–20 min, 5–15 % A; 20–30 min, 15–15 % A; 30–35 min, 15–18 % A; 35–60 min, 18–22 % A. The injection volume was 10.00 μL. The detection wavelength was conducted at 254.0 nm. The phenolic compounds were identified by comparing their retention times (tR) and UV spectra with those of the reference standards and/or the literature data (Qi et al., 2024; Wu et al., 2019). Five major phenolic compounds were quantified using their corresponding standard calibration curves. Their results were expressed as milligrams per gram of dry weight (mg/g DW). of BBL.

### 2.7 Method validation

This study developed a HPLC analytical method and two colorimetric assays for the quantitative determination of five main phenolic compounds, total polyphenols, and total flavonoids, respectively. Method validation was systematically performed for the Folin-Ciocalteu Colorimetry and aluminum chloride (AlCl_3_) Colorimetry assay, evaluating linearity, limit of detection (LOD), limit of quantification (LOQ), precision, spiked recovery rate (accuracy), and solution stability. The LOD and LOQ were calculated using the formulas LOD = 3.3σ/S and LOQ = 10σ/S, where σ represents the standard deviation (SD) of the intercept of the calibration curve, and S denotes the slope of the calibration curve.

For the HPLC-DAD method, a comprehensive development and validation procedure was performed for the simultaneous analysis of five polyphenolic compounds (neochlorogenic acid, chlorogenic acid, rutin, hyperoside, and isoquercitrin). The method validation followed the ICH guidelines (ICH.Q2[R2], 2022) assessed specificity, linearity range, LOD, LOQ, precision, solution stability, method repeatability, and accuracy (Fig. S1, Tables S2–S4). The LOD and LOQ were established using the signal-to-noise (S/N) ratio approach, where the thresholds for the analyte response signal (S) relative to baseline noise (N, representing instrumental background fluctuations) were set at S/N ≥ 3 (LOD) and S/N ≥ 10 (LOQ). Precision, stability, repeatability, and recovery rate were statistically evaluated using the coefficient of variation (CV%) to ensure method reliability and reproducibility.

### 2.8 Assays of different bioactive activities

#### 2.8.1 DPPH radical (DPPHˑ) scavenging activity

The DPPH radical scavenging activity was evaluated according to the previous method (Abderrahim et al., 2013) with slight modifications. Briefly, the sample solution was diluted 100-fold with 50 % ethanol, and 0.10 mL of the diluted solution was mixed with 0.50 mL of DPPH solution (0.10 mmol/L). After incubation in the dark for 30 min, the absorbance was measured at 517 nm using a BioTek Synergy H1 multi-mode microplate reader (BioTek Instruments, USA). A series of Trolox standard solutions prepared using 50 % ethanol were analyzed simultaneously. A blank control was prepared using 50 % ethanol without sample. The results were expressed as Trolox equivalent antioxidant capacity (TEAC) in micromoles per gram of dry weight (μmol TEAC/g DW).

#### 2.8.2 ABTS radical cation (ABTS^+^) scavenging activity

According to the manufacturer's instructions, ABTS^+^ scavenging activity of the was determined using a Total Antioxidant Capacity Assay Kit (ABTS method). The ABTS stock solution was prepared by mixing equal volumes of ABTS solution and oxidation solution, followed by incubation under dark condition at room temperature for 16 h. Prior to analysis, the stock solution was diluted 50-fold with 50 % ethanol (volume ratio of 1:49) to obtain the ABTS working solution. Then 7.00 μL of the 100-fold diluted sample solution was mixed with 280.00 μL ABTS working solution (volume ratio of 1:40) in a 96-well microplate. After reacting in the dark at room temperature for 6 min, the absorbance was immediately measured at 405.00 nm using a microplate reader. A standard curve was constructed using a series of Trolox standard solutions prepared in 50 % ethanol. The 50 % ethanol with ABTS working solution served as blank control. The results were expressed as Trolox equivalent antioxidant capacity (TEAC) in micromoles per gram of dry weight (μmol TEAC/g DW).

#### 2.8.3 α-Glucosidase inhibitory assay

The α-glucosidase inhibitory activity assay was conducted according to the referenced method (Liu et al., 2021) with minor modifications. Briefly, the sample solution was diluted with phosphate buffer (pH 6.8) to obtain the test solution. A mixture containing 200 μL α-glucosidase solution (0.5 U/mL) and 200 μL test solution was incubated at 37.0 °C for 20 min, followed by adding 200 μL 2.5 mmol/L p-nitrophenyl-α-D-glucopyranoside solution. After continuing the reaction at 37.0 °C for another 20 min, the absorbance was measured at 405 nm using a microplate reader. Controls were set as blank control (no enzyme/no sample), negative control (with enzyme/no sample), test sample group (with enzyme/with sample), and sample blank group (no enzyme/with sample). The inhibition rate (%) was calculated from triplicate tests.

#### 2.8.4 Pancreatic lipase inhibitory assay

The pancreatic lipase inhibitory activity was evaluated according to a previously described method (Buchholz and Melzig, 2016) with slight modifications. The sample solution was diluted with phosphate buffer (pH 6.8) to obtain the test solution. A reaction mixture of 50 μL sample and 200 μL pancreatic lipase solution (5 mg/mL, dissolved in 100 mM phosphate buffer, pH 8.2) was incubated at 37 °C for 15 min, followed by adding 50 μL p-nitrophenyl laurate. After further incubation at 37 °C for 45 min, the absorbance was measured at 405 nm using Orlistat as the positive control. The following groups were designed based on the above steps: blank control (no enzyme, no sample), negative control (with enzyme, no sample), test sample group (with enzyme and sample), and sample blank group (no enzyme, with sample). The pancreatic lipase inhibition rate (%) was determined from triplicate tests.

#### 2.8.5 Xanthine oxidase inhibitory assay

The determination of xanthine oxidase inhibitory activity was performed according to the literature method (Kong et al., 2000). The sample solution was diluted with phosphate buffer (pH 6.8) to obtain the test solution. In 140 μL of phosphate buffer (50 mM, pH 7.4), sequentially add 40 μL of xanthine solution (final concentration 150 μM) and 10 μL of the test inhibitor solution (varying concentration gradients). After pre-incubation for 5 min, add 10 μL of XOD enzyme solution (final concentration 0.1 U/mL) to initiate the reaction. The mixture was incubated at 37 °C for 15 min, and the reaction was terminated by adding 50 μL of 1 M HCl. The following groups were designed based on the above steps: blank control (no enzyme, no sample), negative control (with enzyme, no sample), test sample group (with enzyme and sample), and sample blank group (no enzyme, with sample). All experiments were performed in triplicate, and the inhibition rate (%) was calculated.

### 2.9 Statistical analysis

Data were analyzed using IBM SPSS Statistics 27, and results were expressed as mean ± standard error of the mean (SEM). Descriptive statistic included the average value (Mean), standard deviation (SD), median (Me), and the slopes of the regression equations. Principal component analysis (PCA), Orthogonal Partial Least Squares-Discriminant Analysis (OPLS-DA), and hierarchical cluster analysis (HCA) were performed with origin Pro 2019 (Origin-lab Corporation, Northampton, MA, USA). Bar graphs were generated using GraphPad Prism version 9.0 (GraphPad Software, San Diego, CA, USA). In addition, the entropy weight method implemented in SPSSAU was applied for quality assessment of BBL samples.

## 3 Results and discussion

### 3.1 HPLC method validation

In this study, the HPLC method was systematically validated for specificity, linear range, LOD, LOQ, precision, solution stability, method repeatability, and spiked recovery to ensure reliable quantitation of polyphenolic compounds in BBL. By comparing retention times and UV spectra with reference standards, five major phenolic compounds in BBL were identified as neochlorogenic acid (Peak 1), chlorogenic acid (Peak 2), rutin (Peak 3), hyperoside (Peak 4), and isoquercitrin (Peak 5), respectively (Fig. S1 and Table S2). The regression equations and concentration ranges for each compound were as follows: y = 9.9837x − 5.8752 (neochlorogenic acid, 3.90–280.80 μg/mL, *R*^2^ = 1.000), y = 8.8682x + 136.58 (chlorogenic acid, 5.00–5000.00 μg/mL, *R*^2^ = 0.999), y = 17.999x − 2.5865 (rutin, 5.00–240.00 μg/mL, *R*^2^ = 0.999), y = 26.037x − 0.4796 (hyperoside, 3.00–150.00 μg/mL, *R*^2^ = 1.000), y = 23.298x + 3.8956 (isoquercitrin, 5.00–360.00 μg/mL, *R*^2^ = 1.000), where y represents chromatographic peak area and x corresponds to concentration (μg/mL). Method validation results revealed LOD and LOQ ranged from 0.13 to 1.09 μg/mL and 0.41–3.60 μg/mL, respectively. The relative standard deviation (RSD) of precision was between 0.205 % and 0.25 %, and the test solution exhibited good stability within 24 h (RSD: 0.10 %–0.81 %). The method repeatability (independently prepared samples, n = 6) and accuracy of spiked recovery showed RSD values of 0.83 %–1.22 % and 0.13 %–1.61 %, respectively, with recovery rates ranging from 97.05 % to 101.91 %. These results confirm that the method exhibits excellent linearity, sensitivity, and accuracy.

### 3.2 Quantification of phenolic compounds

**Fig. 1:**
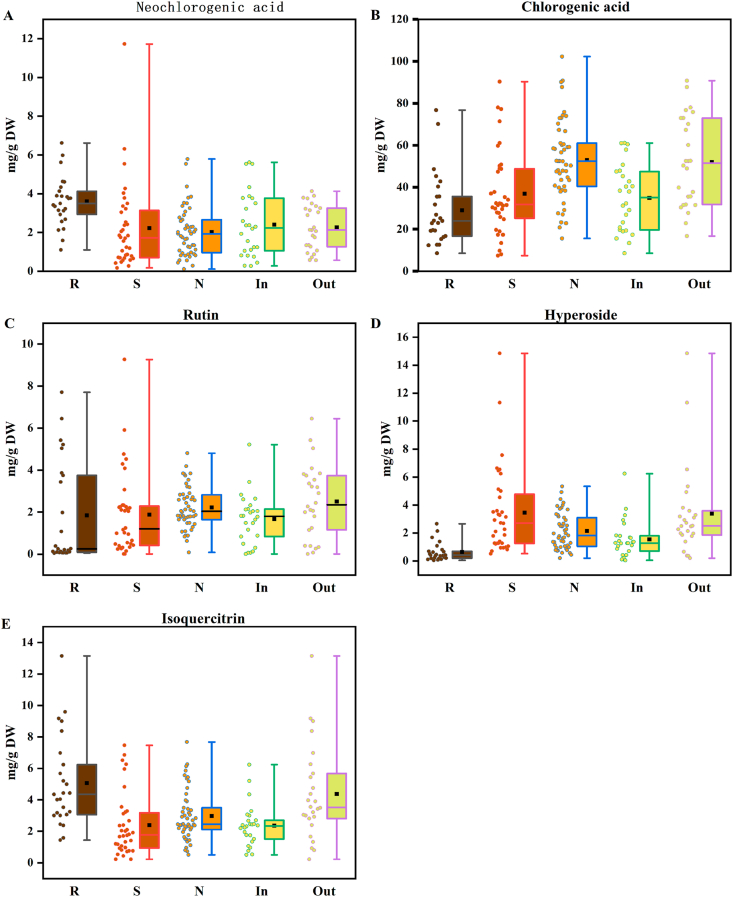
Neochlorogenic acid (A), Chlorogenic acid (B), Rutin (C), Hyperoside (D) and Isoquercitrin(E) content of 110 bathes of BBL extracts represented as Box Plots. (Dark brown), (Red brown), (Orange), (Yellow) and (Yellow green) indicate the rabbiteye (R), southern highbush (S), northern highbush (N), greenhouse cultivation (In) and open-field growth (Out) blueberry samples, respectively.

The contents of the five phenolic compounds in the 110 batches of BBL samples were summarized in Table S1, with comparative profiles across cultivars and growing environments shown in Fig. 1. Among cultivars, rabbiteye BBL exhibited the highest mean levels of neochlorogenic acid (3.20 ± 1.26 mg/g) and isoquercitrin (5.06 ± 2.82 mg/g), while southern highbush cultivars showed highest hyperoside content (3.47 ± 3.06 mg/g). In contrast, northern highbush cultivars accumulated the greatest amount of chlorogenic acid (52.89 ± 19.03 mg/g) and rutin (2.22 ± 1.00 mg/g). With respect to growth environments, neochlorogenic acid levels were comparable between greenhouse and open-field conditions, whereas BBL grown in the open-field conditions contained significantly higher concentrations of chlorogenic acid, rutin, hyperoside, and isoquercitrin than those cultivated in greenhouse.

### 3.3 Folin-Ciocalteu and AlCl_3_ colorimetric method validation

In this study, the Folin-Ciocalteu and AlCl_3_ colorimetric methods were systematically validated through key parameters including specificity, linearity, LOD, LOQ, precision, solution stability, method repeatability, and spiked recovery (Table S3) for the quantitative analysis of TPC and TFC in BBL. For the Folin-Ciocalteu analysis, a standard curve was established by using a series of chlorogenic acid concentrations, affording the regression equation of y = 0.0029x + 0.0821 (R^2^ = 0.9962), indicating excellent linearity across the range of 15.63–250.00 μg/mL. The LOD and LOQ for total polyphenols in BBL were 0.13 μg/mL and 0.43 μg/mL, respectively. The precision (RSD = 1.41 %), repeatability (RSD = 1.74 %), and solution stability (RSD = 0.97 %) all met the criteria for reliable quantification of total polyphenols. Under the chromogenic conditions employing 7.5 % sodium carbonate solution, the absorbance remained stable for 30–180 min (RSD = 0.95 %), and the spiked recovery rate was 101.09 % (RSD = 1.19 %), further confirming the high sensitivity, operational stability, and accuracy for quantitative analysis of total polyphenols in BBL.

In the AlCl_3_ colorimetric assay, a standard curve was established by measuring the absorbance of a series of rutin concentration, giving the regression equation of y = 0.0019x + 0.045 (*R*^2^ = 1), which demonstrate excellent linearity within the range of 15.6–1000.0 μg/mL. Absorbance measurements at 405 nm demonstrated no significant difference between chlorogenic acid/gallic acid and the blank background (ΔA < 0.02), while rutin and BBL samples exhibited characteristic absorption, confirming the method's specificity for flavonoids detection. The method for quantifying total flavonoids in BBL showed a LOD of 0.07 μg/mL and a LOQ of 0.23 μg/mL. Other validation parameters including precision (RSD = 0.77 %), repeatability (n = 6, RSD = 1.39 %), solution stability (24 h, RSD = 1.22 %), chromogenic stability (60 min post-reaction, RSD = 1.22 %), and spiked recovery (mean 98.11 %, RSD = 2.34 %) all complied with accepted analytical standards, confirming its reliability for accurately quantifying total flavonoid in BBL samples.

### 3.4 TPC and TFC in BBL extracts

**Fig. 2:**
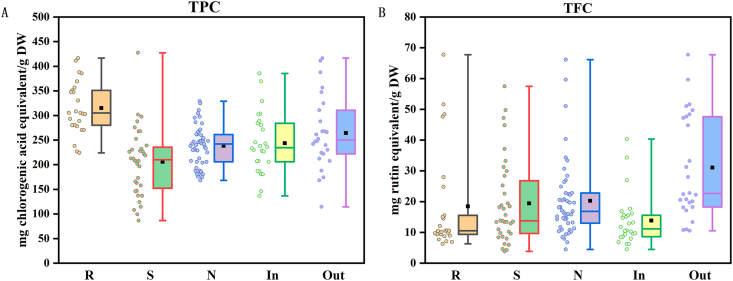
TPC (A), and TFC (B) content of 110 bathes of BBL extracts represented as Box Plots.

A total of 110 BBL samples were analyzed using the optimized methods (Table S1). Based on the data presented in Table S1 and Fig. 2, the content characteristics of total polyphenols and total flavonoids in BBL were evaluated across different varieties and growth environments. Significant differences in TPC were observed among blueberry varieties (one-way ANOVA, *p* < 0.05). Specifically, rabbiteye blueberry (315.19 ± 52.74 mg CAE/g DW) exhibited significantly higher levels compared to northern highbush BBL (238.46 ± 40.35 mg CAE/g DW) and southern highbush BBL (205.83 ± 67.98 mg CAE/g DW). As shown in Fig. 2A, considerable intra-group dispersion was observed across all varieties, indicating substantial variation in total polyphenol content even among subspecies within the same variety. This suggested that genetic background plays a critical role in regulating polyphenol accumulation. TFC showed no significant differences among the three varieties (one-way ANOVA: F = 0.34, *p* = 0.715), with average values ranging from 18.46 to 20.31 mg CAE/g DW. However, both rabbiteye and southern highbush varieties exhibited relatively high variability, whereas northern highbush variety displayed moderate variability. These distinct boxplot patterns highlight varietal differences in the distribution of TFC.

The TPC was slightly higher in BBL from open-field environments (264.58 ± 73.33 mg CAE/g DW) than those from the greenhouse: (243.66 ± 60.87 mg CAE/g DW). Nevertheless, an independent samples *t*-test revealed no significant inter-group difference (*p* = 0.18). Boxplots analysis revealed greater dispersion in the open-field environment group, characterized by a wider box, longer whiskers, and the presence of high-value outliers, indicative of a right-skewed distribution (mean > median). In contrast, the greenhouse environment exhibited a narrower box range and a more homogenous distribution. These findings indicated that open-field environments enhanced variability in TPC across blueberry varieties, while greenhouse conditions promoted more stable polyphenol accumulation under controlled settings. For TFC, significantly higher levels were observed in open-field environments group (31.08 ± 16.56 mg CAE/g DW) compared to greenhouse group (13.84 ± 8.26 mg CAE/g DW). Boxplots revealed a tightly clustered distribution, with narrow whisker ranges under greenhouse group, whereas the open-field environment displayed a markedly elevated box, extended whiskers, and high-value outliers. This distribution pattern suggested that flavonoid accumulation in BBL is dually regulated by both environmental and genetic factors.

### 3.5 Different bioactive activities of BBL extracts

#### 3.5.1 Antioxidant activities of BBL

The antioxidant efficacy of BBL extracts using both DPPH and ABTS radical scavenging assays. The DPPH assay, based on a stable lipophilic free radical, is widely employed to assess the free radical scavenging capacity of plant extracts (Huang et al., 2012). Recognized as a gold standard for comprehensive antioxidant assessment, the ABTS assay provides an indirect measure applicable to both hydrophilic and lipophilic substances (Munteanu and Apetrei, 2021).

**Fig. 3:**
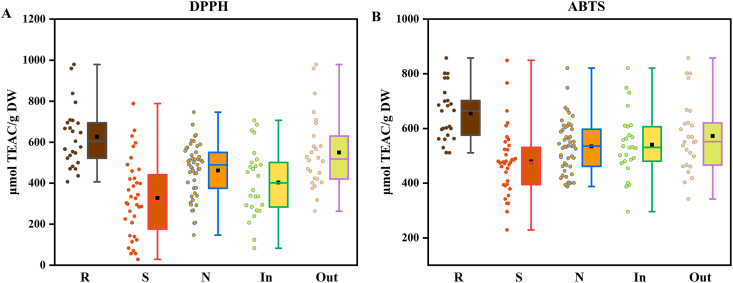
DPPH radical scavenging activity (A), and ABTS radical cation scavenging activity (B) of 110 bathes of BBL extracts represented as Box Plots.

As shown in Table S1 and Fig. 3A, the DPPH assay revealed that the rabbiteye blueberry cultivar R5(out) exhibited the highest antioxidant activity (979.14 μmol TEAC/g DW), while the southern highbush cultivar S21(in) showed the lowest activity (406.76 μmol TEAC/g DW). Among these varieties, rabbiteye demonstrated the highest mean DPPH activity (627.07 ± 150.25 μmol TEAC/g DW), followed by northern highbush (462.20 ± 130.94 μmol TEAC/g DW) and southern highbush (328.04 ± 188.66 μmol TEAC/g DW). Although no significant differences were observed among varieties (P > 0.050). Rabbiteye also showed less variability compared to the other cultivars, suggesting more consistent and superior DPPH radical scavenging activity. When comparing growing environment (Fig. 3A), BBL from open-field environment had higher mean DPPH activity (554.64 ± 182.94 μmol TEAC/g DW) than those of greenhouse conditions (403.87 ± 167.67 μmol TEAC/g DW), indicating superior antioxidant activity in BBL grown under open-field environments.

The highest ABTS values were recorded in rabbiteye R5 (out) (857.78 μmol TEAC/g DW), southern highbush S17(in) (848.89 μmol TEAC/g DW), and northern highbush N24(in) (820.74 μmol TEAC/g DW), all of which were significantly higher than the minimum values within their respective groups (P < 0.05). As illustrated in Fig. 3B, the mean ABTS value of rabbiteye cultivars (654.90 ± 98.79 μmol TEAC/g DW) was significantly greater than those of southern highbush (479.55 ± 123.72 μmol TEAC/g DW) and northern highbush (534.49 ± 95.95 μmol TEAC/g DW) (P < 0.001). Rabbiteye also exhibited lower variability compared to the other two varieties, indicating more consistent and superior ABTS radical scavenging activity in rabbiteye variety. Growing environmental comparisons (Fig. 3B) revealed that BBL from open-field environment showed a slightly higher mean ABTS activity (578.44 ± 129.77 μmol TEAC/g DW) than those from greenhouse conditions (541.32 ± 117.26 μmol TEAC/g DW). Although statistically non-significant, a comparative advantage in ABTS activity was observed in BBL from natural environment.

#### 3.5.2 α-Glucosidase inhibitory activity of BBL extracts

**Fig. 4:**
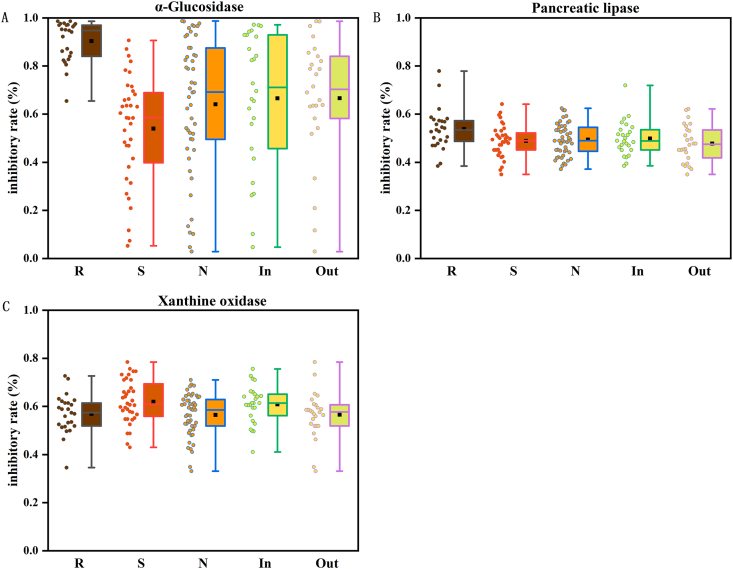
α-Glucosidase inhibitory activity (A), pancreatic lipase inhibitory activity (B), and xanthine oxidase inhibitory activity (C) of 110 BBL extracts represented as Box Plots.

Table S1 and Fig. 4A present the α-glucosidase inhibitory activity data across all BBL samples. Significant differences (P < 0.01) were observed among rabbiteye, southern highbush, and northern highbush cultivars. Rabbiteye BBL exhibited the most potent inhibition (90.33 % ± 8.64 %), significantly surpassing those of southern highbush (53.98 % ± 5.24 %) and northern highbush (64.10 % ± 2.86 %). Among the 25 rabbiteye BBL samples, 16 samples showed inhibition rates exceeding 90.00 %, with the peak value (98.65 %) observed in sample R14(in). The highest activity was recorded in southern highbush BBL sample S28(out) (90.65 %) and northern highbush BBL sample N30(out) (98.73 %), respectively. Environmental comparison (Table S1 and Fig. 4A) revealed comparable α-glucosidase inhibitory activities between BBL from open-field environment (66.59 % ± 28.65 %) and those from greenhouse conditions (66.64 % ± 25.95 %), indicating that environmental factors had minimal influence on this bioactivity.

#### 3.5.3 Pancreatic lipase inhibitory activity of BBL extracts

As shown in Table S1 and Fig. 4B, significant differences (P < 0.05) were observed in pancreatic lipase inhibitory activity among blueberry cultivars. Rabbiteye BBL exhibited the highest overall activity, with sample R4(in) reaching an inhibition rate of 77.90 %–2.03-fold higher than the lowest value within the same cultivar [R18(out): 38.36 %]. Notably, 68 % of rabbiteye BBL samples surpassed 50 % inhibition. Among southern highbush cultivars, the maximum activity was recorded in BBL sample S4(in) (64.13 %), with only 39 % of BBL samples exceeded 50 % inhibition threshold. In northern highbush cultivars, the highest activity was shown in BBL sample N14(in) (62.38 %), and78 % of BBL samples an exhibited inhibition rate above 50 %. Comparative analysis of cultivation environments (Table S1 and Fig. 4B) revealed marginally higher pancreatic lipase inhibition in greenhouse-cultivated BBL (49.84 % ± 7.11 %) than those from open-field environments (47.79 % ± 7.64 %).

#### 3.5.4 Xanthine oxidase inhibitory activity of BBL extracts

Table S1 and Fig. 4C display significant xanthine oxidase inhibition variations among blueberry cultivars (P < 0.05). The mean inhibition rates decreased in the order: southern highbush (62.01 % ± 8.97 %) > rabbiteye (56.92 % ± 8.02 %) > northern highbush (56.43 % ± 8.90 %). The peak inhibition values occurred in samples S16(out) (78.48 %) for southern highbush, R18(in) (72.72 %) for rabbiteye, and N24(in) (71.04 %) for northern highbush. Comparative environmental analysis (Table S1 and Fig. 4C) indicated noticeably higher inhibition in greenhouse-cultivated leaves (60.89 % ± 7.93 %) versus natural-environment BBL (56.56 % ± 9.73 %).

### 3.6 CA and PLS-DA analysis

Cluster analysis (CA), an unsupervised pattern recognition algorithm, automatically groups data into distinct categories based on sample or variable similarities (Everitt, 2004). By reorganizing heatmap rows/columns with integrated dendrograms, this method visually reveals hierarchical relationships among clusters and efficiently identifies high/low-value zones through color gradients, enabling precise localization of homogeneous sample clusters. As a supervised multivariate statistical approach, Partial Least Squares Discriminant Analysis (PLS-DA) projects high-dimensional data into low-dimensional latent spaces to maximize inter-group separation while screening key variables. PLS-DA is widely applied in medical diagnostics, herbal quality control, and food science research (Borràs et al., 2016).

**Fig. 5:**
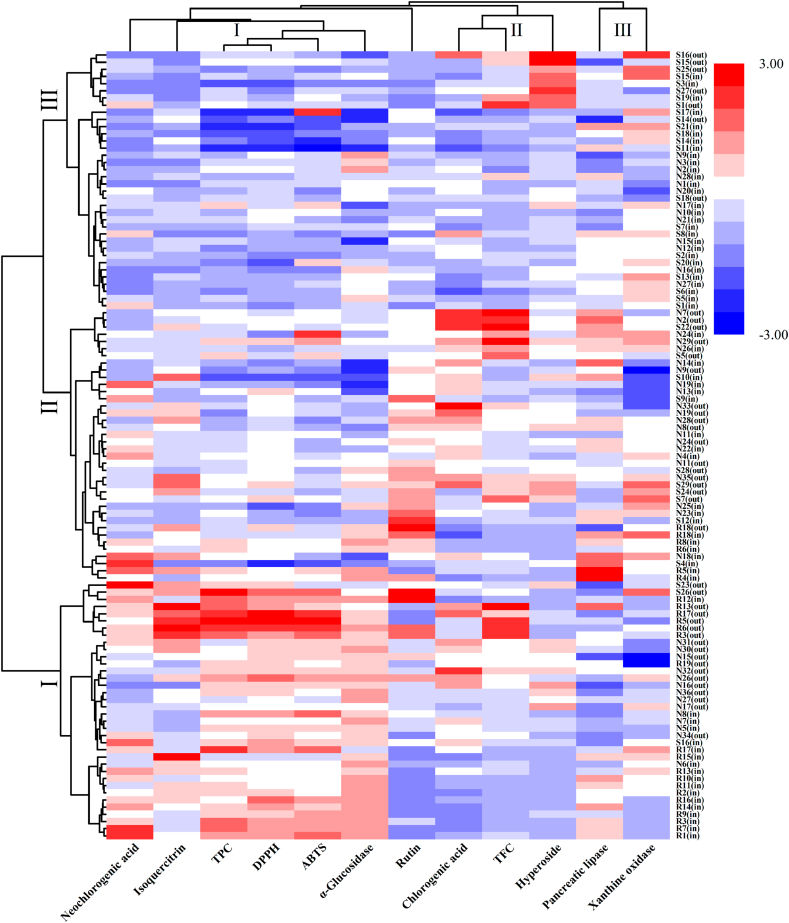
Heat map of hierarchical cluster analysis of quantified polyphenols and different biological assay values in 110 different BBL samples; Color intensity represents the abundance of quantified polyphenols and the strength of biological activities: columns correspond to different samples, rows correspond to quantified polyphenols and measured activity indicators.

CA heatmap based on standardization data from 110 BBL samples revealed three distinct clusters (Fig. 5). Rabbiteye BBL predominantly concentrated in Cluster I (76.00 %, 19/25), with partial distribution in Cluster II (24.00 %, 6/25) and none in Cluster III. Southern highbush BBL were distributed across all three clusters: Cluster I (8.33 %, 3/36), Cluster II (27.78 %, 10/36), and Cluster III (63.89 %, 23/36). Northern highbush BBL were present in Cluster I (28.57 %, 14/49), II (44.90 %, 22/49), and III (26.53 %, 13/49). These results demonstrated significant interspecific divergence between rabbiteye and Southern Highbush, substantial differentiation between rabbiteye and Northern Highbush, yet minimal interspecific disparity between Southern and Northern Highbush. Contrary to the initial hypothesis that each cultivar would form n distinct cluster, the actual clustering pattern was shaped by detected traits, environmental factors, and intraspecific variations among subspecies.

Concurrently, all detected indicators were categorized into three distinct groups: Group I comprised neochlorogenic acid, isoquercitrin, TPC, DPPH radical scavenging capacity, ABTS radical scavenging capacity, and α-glucosidase inhibitory activity; Group II contained rutin, chlorogenic acid, and TFC; Group III included pancreatic lipase inhibitory activity and xanthine oxidase inhibitory activity. This grouping revealed that TPC was closely associated with DPPH/ABTS radical scavenging capacities and α-glucosidase inhibition. Pancreatic lipase and xanthine oxidase inhibitory activities demonstrated contributed comparably to cultivar classification. Chlorogenic acid content was correlated with total flavonoid levels, though the underlying mechanism requires further investigation.

**Fig. 6:**
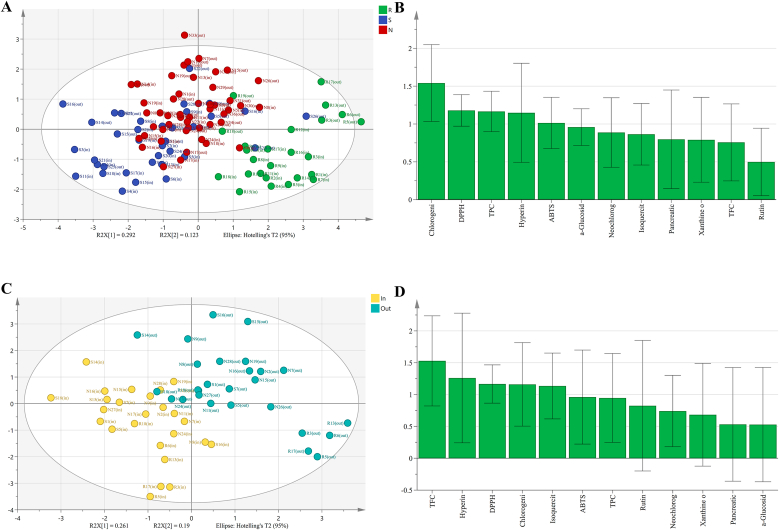
PLS-PLS-DA analysis of BBL. (A)–(B) present PLS-DA score plots and VIP plots for BBL from distinct cultivars; (C)–(D) show corresponding plots for different growing environments, respectively.

PLS-DA was performed on standardized detection data from BBL samples of different cultivars (Fig. 6A and B). The inter-cultivar PLS-DA model demonstrated moderate explanatory power (R^2^X = 0.52, R^2^Y = 0.475) but limited cross-validated predictive ability (Q^2^ = 0.375), indicating fundamental reliability for sample classification. Variables with VIP >1 were prioritized as key contributor cultivar discrimination. The score plot (Fig. 6A) revealed clear separation between rabbiteye and southern/northern highbush cultivars, confirming significant interspecific divergence. Conversely, substantial overlap between Southern and Northern Highbush samples reflected their shared taxonomic clade with relatively minor intraspecific differences. According to the VIP plot (Fig. 6B), the major discriminators of cultivar variation were chlorogenic acid (VIP = 1.542), DPPH radical scavenging capacity (VIP = 1.179), TPC (VIP = 1.165), hyperoside (VIP = 1.147), and ABTS radical scavenging capacity (VIP = 1.013).

PLS-DA was also performed on standardized detection data from the same BBL cultivars grown under different environments (Fig. 6C and D). The inter-environment model exhibited parameters values of R^2^X = 0.451, R^2^Y = 0.683, and Q^2^ = 0.594, indicating satisfactory predictive capability without overfitting risk, thus validating its applicability for sample classification and variable screening. The score plot (Fig. 6C) demonstrated significant separation between open-field and greenhouse-cultivated samples, confirming BBL from these environments possessed substantially distinct metabolic profiles. VIP analysis (Fig. 6D) identified total flavonoids (VIP = 1.539), hyperoside (VIP = 1.260), DPPH radical scavenging capacity (VIP = 1.166), chlorogenic acid (VIP = 1.160), and isoquercitrin (VIP = 1.135) as the key variables contributing to environmental discrimination.

### 3.7 Pearson correlation analysis

**Fig. 7:**
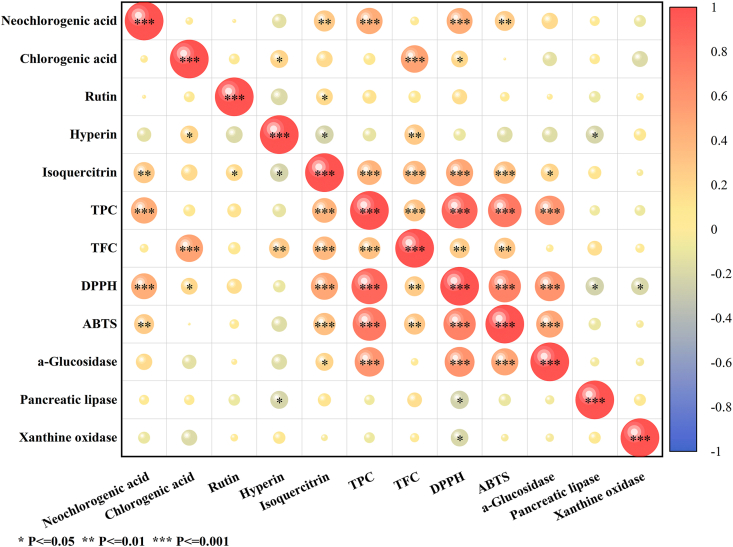
Pearson correlation plot of quantified polyphenols and different biological assay values. Colors represent the magnitude and direction of correlation between variables on each axis.

Pearson correlation analysis was conducted to evaluate the relationship between specific phytochemical constituents and biological activities in BBL (Fig. 7 and Table S4). Correlations were considered significant at *P* > 0.05. Neochlorogenic acid showed significant positive correlations with antioxidant activities (DPPH: R^2^ = 0.450, *P* < 0.001; ABTS: R^2^ = 0.269, *P* < 0.01), while chlorogenic acid was only correlated with DPPH scavenging capacity (R^2^ = 0.191, *P* < 0.05). Isoquercitrin exhibited positive correlations with both antioxidant capacities and α-glucosidase inhibition (DPPH: R^2^ = 0.488, *P* < 0.001; ABTS: R^2^ = 0.337, *P* < 0.01; α-glucosidase: R^2^ = 0.212, *P* < 0.05). Total phenolics demonstrated strong positive correlations with antioxidant activities and α-glucosidase inhibition (DPPH: R^2^ = 0.874, *P* < 0.001; ABTS: R^2^ = 0.763, *P* < 0.001; α-glucosidase: R^2^ = 0.588, *P* < 0.001), and total flavonoids positively correlated with antioxidant capacities (DPPH: R^2^ = 0.272, *P* < 0.01; ABTS: R^2^ = 0.303, *P* < 0.01). Hyperoside showed negative correlation with pancreatic lipase inhibition (R^2^ = −0.218, *P* < 0.05). Among the bioactivities, positive associations between antioxidant capacities were positively correlated with α-glucosidase inhibition (DPPH/α-glucosidase: R^2^ = 0.605, *P* < 0.001; ABTS/α-glucosidase: R^2^ = 0.503, *P* < 0.001), whereas negative correlations between DPPH scavenging capacity was negatively correlated with both pancreatic lipase (R^2^ = −0.229, *P* < 0.05) and xanthine oxidase inhibition (R^2^ = −0.203, *P* < 0.05). This study systematically deciphers compound-specific bioactivity correlations in BBL, but the potential contributions from unidentified compounds warrant further investigation.

### 3.8 Entropy weight method for systematic quality assessment of BBL

The entropy weight method (EWM) is an objective weighting approach that assigns weights to evaluation indicators based on the data dispersion (Nikunj Agarwal, 2015). In this method, a higher variability in indicator values corresponds to lower information entropy, indicating greater information content and consequently a higher assigned weight. In this study, TPC and bioactivity data served as the evaluation indicators for BBL. Applying the EWM to calculate weights and then integrating these weighted values enabled a systematic quality assessment of BBL.

**Fig. 8:**
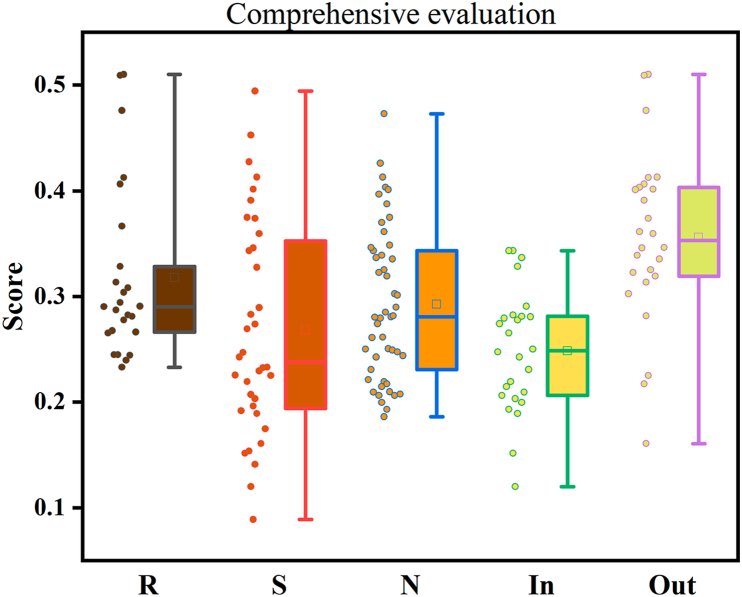
Comprehensive scores of 110 bathes of BBL.

Following forwardization and non-negative translation processing of detection indicators for BBL samples, the EWM was applied to calculate the information entropy (*e*), information utility value (*d*), and weight coefficients (*w*) for each of the 12 indicators. As summarized in Table 1, hyperoside possessed the highest weight (0.169), while xanthine oxidase inhibitory activity showed the lowest weight (0.031). Comprehensive scoring based on these parameters (Table S5) revealed rabbiteye BBL achieved the highest mean score (0.32 ± 0.08), followed by Northern Highbush (0.29 ± 0.07) and Southern Highbush (0.27 ± 0.10) BBL. The top-ranking individual samples was rabbiteye BBL R6 (out) scoring 0.51. Notably, open-field BBL exhibited significantly higher mean scores (0.36 ± 0.08) than greenhouse-cultivated BBL (0.25 ± 0.06) (*P* < 0.05) (Fig. 8).

**Table 1:** Summary of weight calculation Results Using EWM.

	information entropy (*e*)	information utility value (*d*)	weight coefficients (*w*)
Neochlorogenic acid	0.9447	0.0553	10.68 %
Chlorogenic acid	0.9576	0.0424	8.19 %
Rutin	0.9208	0.0792	15.31 %
Hyperin	0.9127	0.0873	16.87 %
Isoquercitrin	0.9447	0.0553	10.69 %
TPC	0.9791	0.0209	4.04 %
TFC	0.9300	0.0700	13.53 %
DPPH	0.9759	0.0241	4.67 %
ABTS	0.9827	0.0173	3.34 %
α-Glucosidase	0.9763	0.0237	4.59 %
Pancreatic lipase	0.9741	0.0259	5.01 %
Xanthine oxidase	0.9841	0.0159	3.08 %

Comprehensive evaluation using EWM confirmed that rabbiteye BBL exhibited superior overall quality compared to Southern and Northern Highbush varieties. In addition, BBL cultivated in open-field environments demonstrating significantly higher quality than those grown under greenhouse conditions. These conclusions are consistent with the analytical results from both chemical constituents (chlorogenic acid, rutin, hyperoside, total phenolics, and total flavonoids) and bioactivities (antioxidant capacity, α-glucosidase inhibition, and pancreatic lipase inhibition), thereby validating the applicability and reliability of EWM for the quality assessment of BBL.

## 4 Discussion

In summary, this study systematically characterized the chemical profiles and bioactivity signatures of BBL across different cultivars and growing environments. Specifically, the optimized HPLC-DAD method achieved efficient separation and accurate quantification of five major compounds including neochlorogenic acid, chlorogenic acid, rutin, hyperoside, and isoquercitrin. In addition, the improved analysis method for TFC demonstrated enhanced specificity and yielded more reliable results, successfully addressing the anomalously high and theoretically inconsistent values reported in previous literature. Application of this methodological system to analyze BBL from different cultivars and growing environments revealed that rabbiteye BBL exhibited superior overall quality compared to southern and northern highbush cultivars, while open-field samples consistently outperformed those from greenhouse conditions. These findings were strongly supported by the comprehensive evaluation through the entropy weight method, which not only validated the reliability of our experimental data but also highlighted the utility and robustness of this approach in quality assessment of BBL. Additionally, CA and PLS-DA analysis were employed to elucidate the variation patterns in chemical composition and bioactivities across different cultivars and growing environments. Subsequent Pearson correlation analysis provided preliminary insights into the intrinsic relationships between specific components and their associated biological activities. Overall, this study established a robust methodological foundation and provides substantial data support for the comprehensive utilization and scientific establishment of quality standards for BBL. Nevertheless, the precise mechanisms linking chemical composition to bioactivity still require further in-depth investigation.

## CRediT authorship contribution statement

Linhang Han: Writing – original draft, Data curation, Formal analysis, Visualization. Shuai Sun: Writing – review & editing, Supervision. Yuqi Yang: Data curation. Yiling Chen: Resources. Gangqiang Dong: Resources. Tingzhao Li: Writing – review & editing. Yiming Li: Writing – review & editing, Funding acquisition, Project administration, Supervision. Liuqiang Zhang: Writing – review & editing, Supervision.

## Declaration of competing interest

The authors declare that they have no known competing financial interests or personal relationships that could have appeared to influence the work reported in this paper.

## Data Availability

Data will be made available on request.
