# Supplementary material for: Comparisons of phenolic components and anti-metabolic activities during different cultivars and growing conditions of blueberry leaves
Source: Curr Res Food Sci. 2025 Nov 24;11:101256. doi: 10.1016/j.crfs.2025.101256 (PMC12704199; doi:10.1016/j.crfs.2025.101256)
Supplement: Multimedia component 1 [file mmc1.docx]

Comparisons of phenolic components and anti-metabolic activities during different cultivars and growing conditions of blueberry leaves

**Authors**

Linhang Han^a,#^, Shuai Sun^b,#^, Yuqi Yang^a^, Yiling Chen^c^, Gangqiang Dong^c^, Tingzhao Li^b,c,*^, Yiming Li^a,*^, Liuqiang Zhang^a,*^.

**Affiliations**

^a^ School of Pharmacy, Shanghai University of Traditional Chinese Medicine, Shanghai, 201203, China

^b^ Amway (Shanghai) Innovation & Science Co., Ltd., Shanghai, 201203, China

^c^ Amway (China) Botanical R&D Center, Wuxi, 214115, China

^#^ These authors contributed equally to this paper.

* Authors to whom correspondence should be addressed: School of Pharmacy, Shanghai University of Traditional Chinese Medicine, No 1200, Cailun Road, Pudong New Area, Shanghai, 201203, China, Email: ymlius@163.com (Y. Li) and 04100217@163.com (L. Zhang); Amway (China) Botanical R&D Center, No. 188, Feifeng Road, Wuxi, 214115, Jiangsu, China, Email: teric.li@amway.com.

Table S1 Values of 12 indicators in 110 BBL samples.

| Sample code | Neochlorogenic acid | Chlorogenic acid | Rutin | Hyperin | Isoquercitrin | TPC | TFC | DPPH | ABTS | α-Glucosidase | Pancreatic lipase | Xanthine oxidase |
| --- | --- | --- | --- | --- | --- | --- | --- | --- | --- | --- | --- | --- |
|  | (mg/g DW) | (mg/g DW) | (mg/g DW) | (mg/g DW) | (mg/g DW) | (mg CA/g DW) | (mg RT/g DW) | (μmol TEAC/g DW) | (μmol TEAC/g DW) | % | % | % |
| Rabbiteye blueberry | | | | | | | | | | | | |
| R1(in) | 5.99 ± 0.06 | 23 ± 0.28 | 0.07 ± 0 | 1.1 ± 0.01 | 3.53 ± 0.03 | 330.21 ± 0.33 | 14.51 ± 0.12 | 652.4 ± 15.9 | 784.44 ± 0.01 | 97.01 ± 0.24 | 57.25 ± 0.14 | 53.28 ± 0.12 |
| R2(in) | 2.57 ± 0.02 | 12.63 ± 0.14 | 0.12 ± 0 | 0.46 ± 0.01 | 4.44 ± 0.04 | 308.14 ± 4.23 | 8.73 ± 0.12 | 552.96 ± 19.25 | 602.59 ± 0.01 | 95.02 ± 0.21 | 52.75 ± 0.15 | 58.8 ± 0.12 |
| R3(in) | 3.87 ± 0.01 | 19.35 ± 0.02 | 1.98 ± 0 | 0.09 ± 0 | 3.01 ± 0.01 | 369.52 ± 4.55 | 6.27 ± 0.12 | 706.86 ± 7.53 | 682.96 ± 0.01 | 97.16 ± 0.24 | 55.72 ± 0.14 | 49.68 ± 0.12 |
| R3(out) | 3.49 ± 0.04 | 35.56 ± 0.39 | 5.03 ± 0.05 | 0.38 ± 0 | 8.37 ± 0.08 | 356.64 ± 10.4 | 48.38 ± 0.21 | 708.04 ± 22.6 | 784.44 ± 0.01 | 87.21 ± 0.12 | 53.4 ± 0.16 | 55.92 ± 0.12 |
| R4(in) | 3.12 ± 0.03 | 12.56 ± 0.07 | 3.86 ± 0.04 | 0.17 ± 0 | 1.58 ± 0.01 | 277.79 ± 7.48 | 7.11 ± 0.12 | 535.8 ± 40.18 | 600 ± 0.01 | 97.84 ± 0.32 | 77.9 ± 0.07 | 57.36 ± 0 |
| R5(in) | 5.62 ± 0.01 | 40.33 ± 0.03 | 0.06 ± 0.01 | 0.24 ± 0.01 | 6.24 ± 0.02 | 280.78 ± 1.95 | 15.49 ± 0.12 | 482.53 ± 30.14 | 607.04 ± 0 | 94.81 ± 0.12 | 71.96 ± 0.09 | 61.44 ± 0.24 |
| R5(out) | 3.15 ± 0.04 | 31.79 ± 0.36 | 0.07 ± 0 | 1.39 ± 0.02 | 9 ± 0.1 | 416.64 ± 1.63 | 47.61 ± 0.12 | 979.14 ± 66.13 | 857.78 ± 0.01 | 80.54 ± 0.61 | 49.26 ± 0.17 | 46.32 ± 0.12 |
| R6(in) | 3.23 ± 0.02 | 26.97 ± 0.06 | 3.44 ± 0.01 | 0.06 ± 0 | 3.07 ± 0.02 | 288.83 ± 7.48 | 9.37 ± 0.12 | 547.04 ± 2.51 | 575.93 ± 0 | 92.21 ± 0 | 47.75 ± 0.17 | 61.44 ± 0.37 |
| R6(out) | 3.89 ± 0.01 | 35.65 ± 0.11 | 5.43 ± 0.01 | 0.37 ± 0 | 9.18 ± 0.05 | 387.68 ± 2.31 | 51.62 ± 0.32 | 837.08 ± 56.08 | 801.11 ± 0.01 | 98.63 ± 0.01 | 45.58 ± 0.18 | 60.72 ± 0.21 |
| R7(in) | 6.62 ± 0.08 | 19.56 ± 0.23 | 0.2 ± 0 | 0.63 ± 0.01 | 3.18 ± 0.03 | 351.13 ± 5.2 | 9.79 ± 0.12 | 668.39 ± 1.67 | 699.63 ± 0.01 | 95.19 ± 0.01 | 57.03 ± 0.14 | 51.6 ± 0.21 |
| R8(in) | 3.83 ± 0.05 | 25.22 ± 0.27 | 3.75 ± 0.03 | 0.13 ± 0 | 4.04 ± 0.05 | 280.09 ± 6.83 | 10.14 ± 0 | 521.59 ± 1.67 | 560.74 ± 0 | 94.17 ± 0.01 | 54.49 ± 0.15 | 62.16 ± 0.21 |
| R9(in) | 2.93 ± 0.02 | 15.35 ± 0.09 | 0.25 ± 0.04 | 1.69 ± 0.02 | 3.25 ± 0.01 | 304.92 ± 6.83 | 7.68 ± 0.24 | 605.05 ± 22.6 | 701.85 ± 0 | 97.6 ± 0.03 | 50.14 ± 0.16 | 53.52 ± 0.12 |
| R10(in) | 3.35 ± 0.02 | 24.1 ± 0.08 | 0.06 ± 0 | 0.81 ± 0.01 | 3.01 ± 0.01 | 270.44 ± 4.88 | 8.94 ± 0.24 | 470.69 ± 13.39 | 599.63 ± 0 | 96.38 ± 2.88 | 57.75 ± 0.14 | 58.8 ± 0.21 |
| R11(in) | 2.18 ± 0.01 | 23.97 ± 0.1 | 0.09 ± 0 | 0.19 ± 0 | 4.99 ± 0.01 | 293.2 ± 20.16 | 9.37 ± 0.12 | 500.28 ± 75.34 | 597.04 ± 0.02 | 96.93 ± 0.28 | 56.81 ± 0.14 | 62.64 ± 0.32 |
| R12(in) | 4.63 ± 0.02 | 19.32 ± 0.03 | 7.71 ± 7.54 | 0.38 ± 0 | 2.39 ± 0 | 347.68 ± 9.43 | 6.9 ± 0.12 | 694.43 ± 15.07 | 689.26 ± 0.01 | 96.96 ± 0.03 | 46.88 ± 0.17 | 51.36 ± 0.24 |
| R13(in) | 4.34 ± 0.02 | 45.23 ± 0.2 | 0.06 ± 0 | 0.43 ± 0.01 | 5.2 ± 0.03 | 303.77 ± 4.55 | 15 ± 0.21 | 469.5 ± 25.11 | 563.33 ± 0.02 | 82.8 ± 0.02 | 48.7 ± 0.17 | 63.12 ± 0.12 |
| R13(out) | 3.76 ± 0 | 70.15 ± 0.13 | 0.38 ± 0 | 0.67 ± 0 | 13.15 ± 0.04 | 347.45 ± 18.21 | 67.75 ± 0.44 | 692.65 ± 34.32 | 664.44 ± 0 | 76.51 ± 0 | 62.14 ± 0.13 | 51.84 ± 2.88 |
| R14(in) | 4.61 ± 0.01 | 16.29 ± 0.11 | 0.17 ± 0.01 | 0.52 ± 0.01 | 4.01 ± 0.01 | 302.85 ± 3.9 | 9.72 ± 0.37 | 666.61 ± 14.23 | 665.93 ± 0.01 | 98.65 ± 0.08 | 58.7 ± 0.13 | 52.56 ± 0.12 |
| R15(in) | 1.59 ± 0 | 12.39 ± 0.03 | 0.04 ± 0 | 0.26 ± 0.01 | 9.59 ± 0.04 | 226.76 ± 2.28 | 10.56 ± 0 | 406.76 ± 10.05 | 511.85 ± 0 | 81.68 ± 0.04 | 54.13 ± 0.15 | 65.28 ± 0 |
| R16(in) | 3.42 ± 0.04 | 22.81 ± 0.01 | 0.11 ± 0 | 0.7 ± 0.01 | 4.44 ± 0.05 | 270.67 ± 0 | 12.11 ± 0.24 | 794.46 ± 15.9 | 685.93 ± 0 | 97.51 ± 0 | 52.73 ± 0.16 | 49.92 ± 0.12 |
| R17(in) | 3.77 ± 0.01 | 42.75 ± 0.14 | 0.12 ± 0 | 0.43 ± 0 | 2.45 ± 0 | 385.38 ± 2.28 | 10.63 ± 1.24 | 647.08 ± 23.44 | 731.11 ± 0 | 65.41 ± 0.24 | 46.94 ± 0.18 | 71.52 ± 0.24 |
| R17(out) | 4.13 ± 0.05 | 76.74 ± 0.52 | 0.26 ± 0 | 2.14 ± 0.02 | 6.98 ± 0.06 | 411.36 ± 0.65 | 27.96 ± 0.61 | 959.01 ± 10.88 | 802.22 ± 0.01 | 84.01 ± 0.21 | 39.37 ± 0.18 | 56.88 ± 0.12 |
| R18(in) | 1.1 ± 0 | 8.58 ± 0.03 | 5.21 ± 0.04 | 0.12 ± 0 | 4.35 ± 0.03 | 238.25 ± 21.13 | 10.49 ± 0.12 | 435.76 ± 24.28 | 529.63 ± 0.01 | 82.4 ± 0.61 | 58.1 ± 0.15 | 72.72 ± 0.12 |
| R18(out) | 2.12 ± 0.02 | 16.81 ± 0.12 | 6.46 ± 0.06 | 0.22 ± 0 | 5.68 ± 0.08 | 224.23 ± 7.15 | 10.56 ± 0 | 565.99 ± 15.9 | 511.48 ± 0.01 | 85.52 ± 0.24 | 38.36 ± 0.19 | 59.52 ± 0.44 |
| R19(out) | 2.68 ± 0.03 | 48.66 ± 0.41 | 1.07 ± 0.01 | 2.66 ± 0.02 | 1.45 ± 0.01 | 305.38 ± 3.58 | 24.79 ± 0.24 | 576.64 ± 9.21 | 562.22 ± 0.01 | 86.15 ± 0.12 | 50.51 ± 0.15 | 34.56 ± 0.32 |
| Southern highbush blueberry | | | | | | | | | | | | |
| S1(in) | 3.78 ± 0.06 | 32.26 ± 0.46 | 0.01 ± 0 | 3.28 ± 0.02 | 0.97 ± 0 | 207.45 ± 21.13 | 6.2 ± 0.12 | 239.25 ± 34.32 | 454.44 ± 0.01 | 66.3 ± 0.23 | 47.25 ± 0.17 | 60.72 ± 0.02 |
| S1(out) | 3.36 ± 0.03 | 31.5 ± 0.34 | 0.01 ± 0 | 6.54 ± 0.07 | 1.34 ± 0.01 | 268.14 ± 2.28 | 49.72 ± 0.24 | 386.04 ± 2.51 | 522.22 ± 0.01 | 63.56 ± 0.22 | 47.2 ± 0.18 | 57.84 ± 0.01 |
| S2(in) | 1.71 ± 0.01 | 50.2 ± 0.4 | 1.01 ± 0.01 | 1.4 ± 0.01 | 1.72 ± 0.01 | 201.93 ± 8.13 | 13.45 ± 0.12 | 207.88 ± 63.62 | 395.56 ± 0.02 | 46.81 ± 0 | 53.04 ± 0.15 | 58.8 ± 0.12 |
| S3(in) | 0.68 ± 0.01 | 32.02 ± 0.07 | 0.14 ± 0 | 6.47 ± 0.02 | 0.44 ± 0 | 137.1 ± 15.93 | 13.73 ± 0.42 | 138.92 ± 7.12 | 390.74 ± 0 | 38.03 ± 0.98 | 48.41 ± 0.17 | 61.2 ± 0.32 |
| S4(in) | 6.31 ± 0.1 | 32.41 ± 0.42 | 0.31 ± 0.01 | 3.33 ± 0.06 | 0.71 ± 0.18 | 146.99 ± 3.25 | 13.45 ± 0.12 | 70.26 ± 24.69 | 354.44 ± 0.02 | 33.05 ± 0.01 | 64.13 ± 0.12 | 62.4 ± 0.12 |
| S5(in) | 2.37 ± 0.01 | 31.21 ± 0.02 | 1.15 ± 0.05 | 1.27 ± 0 | 1.75 ± 0 | 228.14 ± 0.98 | 8.59 ± 0.12 | 333.96 ± 5.86 | 480.74 ± 0.01 | 84.22 ± 0.02 | 50.07 ± 0.16 | 64.08 ± 0.18 |
| S5(out) | 3.23 ± 0 | 49 ± 0.18 | 2.06 ± 0.07 | 2.72 ± 0.01 | 3.22 ± 0.01 | 288.14 ± 14.3 | 47.19 ± 0.32 | 423.33 ± 51.9 | 620.74 ± 0.01 | 63.54 ± 0.21 | 51.17 ± 0.16 | 57.6 ± 0.02 |
| S6(in) | 0.56 ± 0.04 | 9.72 ± 0.01 | 0.54 ± 0.05 | 0.94 ± 0.01 | 2.02 ± 0.01 | 172.97 ± 13.98 | 3.87 ± 0.12 | 326.26 ± 3.35 | 467.59 ± 0.01 | 77.41 ± 0.12 | 50.72 ± 0.17 | 66.24 ± 0.04 |
| S7(in) | 1.56 ± 0.01 | 18.93 ± 0.01 | 2.32 ± 0 | 1.27 ± 0.01 | 1.07 ± 0.01 | 207.22 ± 15.61 | 7.96 ± 0.24 | 366.51 ± 16.74 | 504.07 ± 0 | 58.3 ± 0 | 42.03 ± 0.2 | 59.52 ± 0.02 |
| S7(out) | 3.04 ± 0.04 | 37.68 ± 0.53 | 4.08 ± 0.06 | 3.6 ± 0.02 | 2.4 ± 0.02 | 268.14 ± 16.58 | 44.79 ± 0.21 | 579.6 ± 5.02 | 569.26 ± 0 | 65.79 ± 0.12 | 45.29 ± 0.18 | 73.2 ± 0.01 |
| S8(in) | 3.49 ± 0.01 | 71.38 ± 0.37 | 0.26 ± 0 | 1.28 ± 0.01 | 0.81 ± 0.04 | 163.08 ± 5.2 | 13.87 ± 0.12 | 344.02 ± 20.09 | 407.22 ± 0 | 46.9 ± 0.68 | 56.66 ± 0.15 | 65.04 ± 0.12 |
| S9(in) | 4.27 ± 0.01 | 35.32 ± 0.32 | 4.77 ± 0.03 | 1.23 ± 0.02 | 2.11 ± 0.02 | 196.64 ± 22.76 | 9.79 ± 0.12 | 459.44 ± 22.6 | 472.59 ± 0.01 | 58.44 ± 0.03 | 43.96 ± 0.19 | 42.96 ± 0.21 |
| S10(in) | 0.83 ± 0 | 59.7 ± 0 | 2.25 ± 0 | 3.5 ± 0 | 6.86 ± 0 | 124 ± 0.65 | 5.92 ± 0.21 | 113.77 ± 2.51 | 326.48 ± 0 | 24.87 ± 0.02 | 58.71 ± 0.14 | 44.4 ± 0.61 |
| S11(in) | 0.48 ± 0 | 7.4 ± 0.02 | 0.95 ± 0 | 0.95 ± 0 | 2.05 ± 0 | 86.53 ± 2.28 | 4.65 ± 0.37 | 56.44 ± 4.19 | 228.89 ± 0.01 | 7.36 ± 0.01 | 55.99 ± 0.15 | 55.2 ± 0.42 |
| S12(in) | 0.86 ± 0 | 25.79 ± 0.12 | 5.9 ± 0.02 | 2.11 ± 0.01 | 1.81 ± 0 | 232.28 ± 13.98 | 15.63 ± 0.21 | 294.3 ± 11.72 | 476.3 ± 0 | 49.49 ± 0.21 | 54.08 ± 0.16 | 52.56 ± 0.12 |
| S13(in) | 0.69 ± 0.01 | 17.36 ± 0.12 | 2.33 ± 0.01 | 2.7 ± 0.02 | 3.12 ± 0.02 | 207.91 ± 5.53 | 10.21 ± 0.12 | 302.58 ± 3.35 | 477.04 ± 0.01 | 71.93 ± 0.21 | 48.37 ± 0.18 | 67.68 ± 2.88 |
| S14(in) | 0.28 ± 0.01 | 13.42 ± 0.23 | 2.14 ± 0.03 | 1.27 ± 0.01 | 3.29 ± 0.04 | 146.07 ± 5.85 | 8.38 ± 0.32 | 123.83 ± 10.05 | 295.93 ± 0.02 | 26.87 ± 0.32 | 45.1 ± 0.19 | 66 ± 0.12 |
| S14(out) | 0.72 ± 0 | 27.73 ± 0.02 | 2.1 ± 0.02 | 1.86 ± 0.01 | 3.55 ± 0 | 114.34 ± 23.41 | 10.99 ± 0.21 | 263.52 ± 18.42 | 342.22 ± 0.01 | 11.64 ± 0 | 34.94 ± 0.2 | 54.72 ± 0 |
| S15(in) | 1.24 ± 0 | 19.72 ± 0.05 | 0.3 ± 0 | 6.25 ± 0.03 | 0.76 ± 0.01 | 226.87 ± 2.11 | 12.89 ± 0 | 284.24 ± 9.21 | 486.3 ± 0 | 69.59 ± 0.19 | 51.29 ± 0.17 | 75.6 ± 1.95 |
| S15(out) | 2.14 ± 0.01 | 30.35 ± 0.23 | 0.33 ± 0.01 | 14.84 ± 0.11 | 0.92 ± 0.01 | 252.51 ± 30.89 | 33.24 ± 0.12 | 523.96 ± 20.09 | 551.11 ± 0.01 | 63.18 ± 0.16 | 37.82 ± 0.19 | 58.32 ± 0.98 |
| S16(in) | 5.54 ± 0.12 | 61.06 ± 1.24 | 2.04 ± 0.05 | 1.8 ± 0.04 | 2.67 ± 0.06 | 301.93 ± 0 | 17.68 ± 0.12 | 657.14 ± 10.88 | 610.19 ± 0 | 87.08 ± 0.16 | 42.38 ± 0.2 | 60.48 ± 0.01 |
| S16(out) | 0.57 ± 0 | 77.93 ± 0.24 | 0.4 ± 0 | 11.32 ± 0.05 | 0.23 ± 0 | 212.28 ± 5.85 | 57.47 ± 0.21 | 401.43 ± 17.58 | 440 ± 0 | 20.86 ± 0.15 | 49.8 ± 0.15 | 78.48 ± 0.26 |
| S17(in) | 0.17 ± 0 | 8.12 ± 0.12 | 2.25 ± 0 | 1 ± 0 | 2.37 ± 0.01 | 108.14 ± 1.63 | 4.3 ± 0.12 | 56.94 ± 9.21 | 848.89 ± 0.01 | 5.24 ± 4.17 | 42.93 ± 0.2 | 71.76 ± 0.01 |
| S18(in) | 1.05 ± 0 | 17.41 ± 0.14 | 0.49 ± 0 | 0.72 ± 0.01 | 0.54 ± 0.01 | 136.87 ± 1.95 | 9.58 ± 0.61 | 82.39 ± 6.7 | 394.26 ± 0.02 | 31.31 ± 0.03 | 51.31 ± 0.16 | 66.24 ± 0.01 |
| S18(out) | 1.84 ± 0 | 31.52 ± 0.06 | 1.25 ± 0 | 2.93 ± 0 | 1.67 ± 0.01 | 222.16 ± 10.73 | 21.13 ± 0.21 | 420.38 ± 12.56 | 460.74 ± 0.01 | 71.46 ± 0.02 | 45.05 ± 0.16 | 48.72 ± 0.02 |
| S19(in) | 1.75 ± 0.03 | 29.56 ± 0.5 | 0.24 ± 0 | 6.64 ± 0.1 | 0.76 ± 0.02 | 230.9 ± 8.78 | 37.12 ± 0.12 | 298.44 ± 14.23 | 559.63 ± 0 | 48.32 ± 0.02 | 48.55 ± 0.17 | 56.64 ± 0.06 |
| S20(in) | 0.78 ± 0 | 27.99 ± 0.01 | 0.65 ± 0.02 | 0.82 ± 0.01 | 1.42 ± 0.01 | 165.61 ± 0.33 | 19.44 ± 0.42 | 143.95 ± 18.42 | 664.07 ± 0.03 | 55.97 ± 0 | 53.15 ± 0.16 | 63.6 ± 0.01 |
| S21(in) | 0.42 ± 0 | 37.04 ± 0.27 | 0.99 ± 0.01 | 0.52 ± 0.01 | 1.19 ± 0.01 | 99.4 ± 10.08 | 18.8 ± 0.21 | 27.82 ± 13.23 | 326.3 ± 0.03 | 53.21 ± 0.01 | 59.66 ± 0.13 | 71.04 ± 0.01 |
| S22(out) | 1.19 ± 0 | 90.3 ± 0.17 | 2.2 ± 0 | 2.27 ± 0.01 | 4.82 ± 0.01 | 219.17 ± 6.5 | 57.47 ± 0.21 | 467.14 ± 31.81 | 537.78 ± 0.01 | 68.22 ± 0.24 | 60.65 ± 0.13 | 59.76 ± 2.6 |
| S23(out) | 11.73 ± 0.11 | 47.35 ± 0.47 | 2.23 ± 0.02 | 3.51 ± 0.03 | 6.52 ± 0.05 | 297.79 ± 10.4 | 18.45 ± 0.12 | 629.91 ± 72.83 | 485 ± 0.03 | 60.44 ± 0.32 | 36.73 ± 0.19 | 54.48 ± 0.21 |
| S24(out) | 2.53 ± 0 | 24.74 ± 0.01 | 4.52 ± 0 | 4.55 ± 0 | 5.96 ± 0 | 212.28 ± 5.85 | 28.38 ± 0.32 | 397.88 ± 4.19 | 474.26 ± 0.02 | 63.07 ± 0.12 | 45.13 ± 0.17 | 71.04 ± 0.24 |
| S25(out) | 1.85 ± 0.01 | 29.92 ± 0.08 | 0.42 ± 0 | 5.13 ± 0.01 | 1.92 ± 0 | 157.56 ± 11.7 | 13.52 ± 0.21 | 286.6 ± 9.21 | 378.89 ± 0.01 | 41.49 ± 0.05 | 47.9 ± 0.16 | 74.64 ± 0.03 |
| S26(out) | 4.04 ± 0.01 | 34.02 ± 0.06 | 9.27 ± 0.01 | 1.06 ± 0.01 | 6.27 ± 0.02 | 427.45 ± 0 | 25.21 ± 0.24 | 787.95 ± 35.16 | 766.67 ± 0 | 78.31 ± 0.28 | 40.14 ± 0.18 | 73.2 ± 0.11 |
| S27(out) | 0.65 ± 0.01 | 48.72 ± 0.13 | 0.48 ± 0 | 7.57 ± 0.05 | 0.22 ± 0 | 238.94 ± 0 | 22.61 ± 0 | 225.64 ± 55.25 | 489.63 ± 0 | 58.43 ± 0.02 | 49.41 ± 0.15 | 48.72 ± 0.03 |
| S28(out) | 2.06 ± 0.05 | 50.88 ± 1.18 | 4.29 ± 0.1 | 3.16 ± 0.08 | 1.21 ± 0.03 | 226.53 ± 2.6 | 18.17 ± 0 | 490.81 ± 8.37 | 480 ± 0 | 90.65 ± 2.6 | 50.44 ± 0.15 | 54.72 ± 0.01 |
| S29(out) | 2.42 ± 0.01 | 77.21 ± 0.06 | 3.06 ± 0 | 5 ± 0.01 | 7.47 ± 0.01 | 275.72 ± 2.6 | 29.93 ± 0.12 | 596.76 ± 17.58 | 523.7 ± 0.01 | 81.98 ± 0.01 | 49.76 ± 0.15 | 74.64 ± 0.12 |
| Northern highbush blueberry | | | | | | | | | | | | |
| N1(in) | 0.56 ± 0 | 52.38 ± 0.09 | 2.72 ± 0.01 | 0.58 ± 0 | 0.73 ± 0 | 212.74 ± 14.3 | 9.37 ± 0.12 | 414.46 ± 7.53 | 537.22 ± 0 | 73.19 ± 0.47 | 48.59 ± 0.18 | 48 ± 2.88 |
| N2(in) | 0.81 ± 0.01 | 47.88 ± 0.3 | 1.48 ± 0.01 | 1.43 ± 0.01 | 1.35 ± 0.01 | 245.84 ± 0 | 4.51 ± 2.88 | 500.88 ± 10.88 | 532.59 ± 0 | 94.32 ± 0.24 | 42.72 ± 0.2 | 49.92 ± 0.21 |
| N2(out) | 0.88 ± 0.02 | 90.73 ± 0.49 | 2.43 ± 0.01 | 2.48 ± 0.03 | 2.81 ± 0.03 | 248.37 ± 6.18 | 51.06 ± 0.12 | 458.85 ± 8.37 | 549.63 ± 0.01 | 63.78 ± 0.12 | 61.64 ± 0.13 | 58.8 ± 0.32 |
| N3(in) | 0.12 ± 0 | 38.66 ± 0.14 | 0.63 ± 0.01 | 1.62 ± 0 | 0.95 ± 0.01 | 229.06 ± 9.43 | 7.96 ± 0.32 | 447.01 ± 0 | 533.7 ± 0.01 | 87.49 ± 0.17 | 41.82 ± 0.2 | 54.24 ± 0.24 |
| N4(in) | 4.37 ± 0.07 | 56.62 ± 0.89 | 1.73 ± 0.02 | 0.99 ± 0.01 | 2.28 ± 0.02 | 247.22 ± 23.41 | 14.44 ± 0.32 | 472.46 ± 5.86 | 491.11 ± 0.01 | 70.67 ± 0.24 | 49.14 ± 0.17 | 64.56 ± 0.05 |
| N5(in) | 1.68 ± 0.01 | 43.51 ± 0.14 | 1.77 ± 0.01 | 1.38 ± 0.01 | 1.5 ± 0 | 297.56 ± 23.08 | 14.3 ± 0.12 | 605.64 ± 41.85 | 609.26 ± 0.01 | 87.52 ± 0.32 | 44.51 ± 0.19 | 53.52 ± 0.08 |
| N6(in) | 1.65 ± 0.01 | 27.05 ± 0.07 | 1.08 ± 0.01 | 0.22 ± 0.05 | 5.36 ± 0.02 | 267.45 ± 13 | 13.03 ± 0.12 | 518.63 ± 34.32 | 563.7 ± 0.01 | 95.74 ± 0.21 | 44.79 ± 0.19 | 60.96 ± 0.28 |
| N7(in) | 2.36 ± 0 | 57.96 ± 0.14 | 1.49 ± 0.01 | 1.69 ± 0.01 | 1.76 ± 0 | 271.24 ± 12.19 | 14.79 ± 0.21 | 515.67 ± 38.51 | 568.52 ± 0.02 | 96.96 ± 0.16 | 45 ± 0.19 | 56.16 ± 0.01 |
| N7(out) | 1.27 ± 0 | 87.75 ± 0.63 | 2.27 ± 0.02 | 1.79 ± 0 | 3.9 ± 0.03 | 266.3 ± 19.18 | 59.65 ± 0.12 | 449.97 ± 0.84 | 569.26 ± 0 | 58.24 ± 0.05 | 58.74 ± 0.14 | 51.84 ± 0.61 |
| N8(in) | 2.19 ± 0.96 | 40.42 ± 30.84 | 2.13 ± 0.26 | 1.07 ± 0.28 | 1.51 ± 0.63 | 329.06 ± 11.7 | 15.56 ± 0.12 | 685.55 ± 20.93 | 748.15 ± 0 | 92.97 ± 0.11 | 39.41 ± 0.21 | 54 ± 0.03 |
| N8(out) | 2.26 ± 0.02 | 60.13 ± 0.48 | 2.9 ± 0.03 | 3.81 ± 0.03 | 3.97 ± 0.05 | 187.91 ± 10.4 | 22.82 ± 0 | 375.39 ± 19.25 | 466.67 ± 0 | 33.23 ± 0 | 55.02 ± 0.14 | 60.96 ± 2.21 |
| N9(in) | 0.75 ± 0 | 23.3 ± 0.16 | 1.64 ± 0.01 | 1.66 ± 0.01 | 2.43 ± 0.02 | 180.78 ± 10.08 | 6.76 ± 0 | 478.97 ± 10.05 | 535.19 ± 0.01 | 96.59 ± 0.09 | 38.51 ± 0.21 | 50.4 ± 0.02 |
| N9(out) | 0.93 ± 0 | 52.51 ± 0.21 | 3.22 ± 0.01 | 1.99 ± 0 | 3.43 ± 0.02 | 168.14 ± 0 | 10.77 ± 0 | 317.97 ± 6.7 | 402.59 ± 0.01 | 2.86 ± 0.12 | 53.07 ± 0.14 | 33.12 ± 0.02 |
| N10(in) | 0.96 ± 0.01 | 46.68 ± 0.14 | 0.89 ± 0.01 | 0.75 ± 0 | 2.32 ± 0.01 | 205.84 ± 17.56 | 10.92 ± 0.12 | 536.98 ± 15.07 | 604.81 ± 0.02 | 49.67 ± 0.14 | 42.1 ± 0.2 | 62.4 ± 0.07 |
| N11(in) | 3.51 ± 0.01 | 50.75 ± 0.02 | 2.63 ± 0.01 | 0.88 ± 0 | 2.31 ± 0 | 243.08 ± 9.75 | 16.13 ± 0.68 | 489.04 ± 14.23 | 558.15 ± 0.02 | 41.49 ± 0.35 | 46.17 ± 0.18 | 61.44 ± 1.67 |
| N11(out) | 2.89 ± 0.01 | 52.54 ± 0.17 | 3.36 ± 0.01 | 2.52 ± 0.01 | 3.01 ± 0.01 | 239.86 ± 22.11 | 20.78 ± 0.12 | 550 ± 11.72 | 597.41 ± 0 | 78.78 ± 0.12 | 47.78 ± 0.16 | 58.56 ± 0 |
| N12(in) | 1.44 ± 0 | 47.64 ± 0.04 | 1.79 ± 0 | 0.73 ± 0 | 2.13 ± 0 | 176.18 ± 8.78 | 8.45 ± 0.21 | 351.12 ± 16.74 | 461.11 ± 0 | 52.29 ± 0.12 | 50.38 ± 0.17 | 62.4 ± 0.02 |
| N13(in) | 3.08 ± 0.02 | 59.07 ± 0.26 | 2.1 ± 0.01 | 0.78 ± 0.01 | 2.11 ± 0.01 | 260.09 ± 28.61 | 15.92 ± 0.32 | 590.85 ± 30.97 | 594.44 ± 0.01 | 16.16 ± 0.36 | 40.65 ± 0.2 | 42.24 ± 0.08 |
| N14(in) | 0.82 ± 0.01 | 74.21 ± 0.18 | 2.85 ± 0.01 | 0.95 ± 0 | 3.18 ± 0.02 | 204 ± 21.46 | 16.55 ± 0.24 | 204.92 ± 22.6 | 422.96 ± 0 | 10.7 ± 0.06 | 62.38 ± 0.13 | 44.88 ± 0.42 |
| N15(in) | 1.23 ± 0.02 | 37.93 ± 0.01 | 2.6 ± 0.01 | 1.12 ± 0.01 | 2.47 ± 0.02 | 230.44 ± 0.98 | 10.28 ± 0.12 | 267.66 ± 12.56 | 473.33 ± 0.01 | 10.19 ± 0.01 | 53.54 ± 0.16 | 60.72 ± 0 |
| N15(out) | 1.75 ± 0 | 52.64 ± 0.23 | 3.54 ± 0.02 | 2.51 ± 0.02 | 3.51 ± 0.01 | 261.47 ± 5.85 | 20.14 ± 0.12 | 580.78 ± 5.02 | 617.41 ± 0 | 92.29 ± 0.03 | 39.06 ± 0.18 | 34.8 ± 0.08 |
| N16(in) | 0.28 ± 0.02 | 29.14 ± 0.11 | 1.82 ± 0.01 | 2.67 ± 0.02 | 0.51 ± 0 | 186.3 ± 2.93 | 9.72 ± 0 | 207.88 ± 1.67 | 400.37 ± 0.01 | 92.93 ± 0.12 | 52.59 ± 0.16 | 59.52 ± 0.01 |
| N16(out) | 0.57 ± 0 | 73.05 ± 0.1 | 2 ± 0 | 5.34 ± 0.01 | 0.8 ± 0.01 | 310.67 ± 1.95 | 19.72 ± 0.12 | 629.91 ± 25.95 | 610.93 ± 0.02 | 82.07 ± 0.01 | 37.12 ± 0.19 | 52.8 ± 0.04 |
| N17(in) | 1.93 ± 0 | 20.9 ± 0.04 | 0.87 ± 0.01 | 3.73 ± 0.01 | 2.25 ± 0.01 | 284 ± 3.25 | 11.76 ± 0.32 | 453.52 ± 12.56 | 611.85 ± 0.01 | 26.18 ± 0.02 | 48.37 ± 0.18 | 64.32 ± 0 |
| N17(out) | 2.06 ± 0.01 | 22.8 ± 0.19 | 1.15 ± 0 | 4.93 ± 2.21 | 2.92 ± 0.02 | 247.45 ± 0.98 | 17.11 ± 0 | 511.53 ± 17.58 | 552.22 ± 0.02 | 78.79 ± 0.24 | 41.79 ± 0.18 | 64.56 ± 0.01 |
| N18(in) | 5.79 ± 0.02 | 59.18 ± 0.08 | 2.71 ± 0.01 | 0.78 ± 0.03 | 5.86 ± 0.02 | 261.93 ± 10.4 | 20.42 ± 0.24 | 516.86 ± 20.09 | 445.19 ± 0 | 13.35 ± 0.06 | 61.63 ± 0.13 | 69.12 ± 0.72 |
| N19(in) | 5.54 ± 0.01 | 61.01 ± 0.02 | 2.04 ± 0.01 | 1.82 ± 0 | 2.7 ± 0.01 | 180.32 ± 47.14 | 13.24 ± 0.24 | 335.14 ± 76.18 | 387.41 ± 0.02 | 4.7 ± 0.42 | 48.98 ± 0.18 | 41.04 ± 0.82 |
| N19(out) | 3.8 ± 0.01 | 75.84 ± 0.19 | 3.74 ± 0.02 | 3.1 ± 0.02 | 4.75 ± 0.03 | 174.57 ± 0 | 22.47 ± 0.12 | 507.39 ± 5.02 | 498.89 ± 0 | 51.78 ± 1.14 | 44.12 ± 0.17 | 51.84 ± 0.52 |
| N20(in) | 2.66 ± 0.03 | 48.07 ± 0.5 | 1.06 ± 0.01 | 2.64 ± 0.01 | 1.45 ± 0.01 | 192.97 ± 0.65 | 10.35 ± 0 | 377.17 ± 6.7 | 393.52 ± 0.01 | 67.07 ± 0.2 | 48.98 ± 0.18 | 42.72 ± 0.44 |
| N21(in) | 1.81 ± 0.08 | 27.61 ± 1.34 | 1.28 ± 0.06 | 2.19 ± 0.11 | 2.4 ± 0.08 | 232.16 ± 7.64 | 11.34 ± 0.12 | 468.32 ± 25.11 | 432.59 ± 0.01 | 36.28 ± 0.33 | 45.17 ± 0.19 | 59.52 ± 0.24 |
| N22(in) | 3.53 ± 0.04 | 58.24 ± 0.35 | 2.04 ± 0 | 1.16 ± 0.01 | 2.44 ± 0.01 | 242.16 ± 2.6 | 14.3 ± 0.12 | 491.4 ± 34.32 | 427.41 ± 0.01 | 53.25 ± 0.02 | 55.1 ± 0.16 | 60.96 ± 0.12 |
| N23(in) | 2.29 ± 0.02 | 44.71 ± 0.48 | 4.81 ± 0.06 | 1 ± 0 | 1.65 ± 0.01 | 246.99 ± 15.28 | 15.78 ± 1.16 | 304.36 ± 14.23 | 398.52 ± 0.01 | 49.51 ± 0.12 | 57.07 ± 0.15 | 63.84 ± 0.01 |
| N24(in) | 2.59 ± 0.02 | 47.44 ± 0.2 | 2.83 ± 0.01 | 1.36 ± 0.02 | 2.4 ± 0.02 | 236.18 ± 27.96 | 34.37 ± 0.44 | 265.29 ± 15.9 | 820.74 ± 0.02 | 45.62 ± 5 | 59.24 ± 0.14 | 71.04 ± 0 |
| N24(out) | 3.26 ± 0.01 | 50.35 ± 0.18 | 3.18 ± 0.01 | 3.19 ± 0.01 | 3.05 ± 0.01 | 230.21 ± 1.95 | 18.24 ± 0.44 | 469.5 ± 11.72 | 461.85 ± 0 | 69.06 ± 0.78 | 55.98 ± 0.13 | 62.88 ± 0.12 |
| N25(in) | 0.89 ± 0.01 | 32.26 ± 0.25 | 4.18 ± 0.03 | 0.57 ± 0 | 1.13 ± 0.01 | 180.55 ± 7.8 | 24.86 ± 0.32 | 146.91 ± 4.19 | 401.11 ± 0 | 83.78 ± 0.03 | 49.43 ± 0.17 | 68.4 ± 0 |
| N26(in) | 2.29 ± 0.03 | 60.48 ± 0.72 | 1.78 ± 0.02 | 2.93 ± 0.03 | 2.39 ± 0.03 | 233.43 ± 13.65 | 40.36 ± 0.21 | 338.1 ± 0 | 528.15 ± 0 | 55.87 ± 0.16 | 56.55 ± 0.14 | 64.08 ± 1.95 |
| N26(out) | 1.35 ± 0 | 72.9 ± 0.29 | 3.81 ± 0.03 | 1.97 ± 0.02 | 5.45 ± 0.04 | 324.92 ± 3.25 | 22.26 ± 0.24 | 746.52 ± 3.35 | 668.15 ± 0.01 | 98.54 ± 0.08 | 45.05 ± 0.16 | 65.28 ± 0.98 |
| N27(in) | 0.44 ± 0 | 15.64 ± 0.04 | 0.83 ± 0 | 1.29 ± 0 | 1.96 ± 0 | 187.45 ± 3.25 | 16.83 ± 0.32 | 292.52 ± 27.62 | 491.85 ± 0 | 72.73 ± 0.03 | 51.59 ± 0.16 | 65.04 ± 0.98 |
| N27(out) | 1.07 ± 0 | 39.87 ± 0.03 | 1.8 ± 0.01 | 3.28 ± 0.01 | 4.37 ± 0.02 | 242.16 ± 2.6 | 18.1 ± 0.24 | 528.1 ± 5.86 | 538.15 ± 0.01 | 96.59 ± 0.02 | 47.74 ± 0.16 | 56.4 ± 2.6 |
| N28(in) | 0.82 ± 0.01 | 38.36 ± 0.19 | 1.82 ± 0.01 | 1.38 ± 0 | 2.21 ± 0.01 | 206.07 ± 6.18 | 26.9 ± 0.24 | 294.3 ± 15.07 | 502.22 ± 0 | 62.31 ± 0.08 | 54.49 ± 0.15 | 52.8 ± 0.03 |
| N28(out) | 1.33 ± 0 | 67.31 ± 0.01 | 3.85 ± 0 | 2.28 ± 0.01 | 6.27 ± 0 | 207.45 ± 6.18 | 20.56 ± 0.24 | 403.8 ± 17.58 | 417.96 ± 0.01 | 54.16 ± 0.03 | 56.06 ± 0.13 | 58.56 ± 0.05 |
| N29(out) | 1.94 ± 0.01 | 74.04 ± 0.11 | 2.56 ± 0 | 4 ± 0.02 | 2.84 ± 0.01 | 304 ± 2.28 | 66.13 ± 7.16 | 574.27 ± 34.32 | 675.93 ± 0 | 69.21 ± 0.02 | 56.97 ± 0.14 | 68.64 ± 0.02 |
| N30(out) | 2.77 ± 0 | 58.46 ± 0.08 | 1.43 ± 0 | 4.42 ± 0.02 | 5.57 ± 0 | 252.74 ± 7.15 | 22.82 ± 0 | 588.48 ± 12.56 | 657.22 ± 0.01 | 98.73 ± 3.11 | 50.11 ± 0.15 | 48.24 ± 0.02 |
| N31(out) | 3.37 ± 0.01 | 66.72 ± 0.22 | 1.64 ± 0.02 | 4.18 ± 0.01 | 6.14 ± 0.04 | 225.15 ± 6.5 | 26.34 ± 0.12 | 568.35 ± 35.99 | 630 ± 0.01 | 84.56 ± 0.14 | 53.38 ± 0.14 | 48.96 ± 0.01 |
| N32(out) | 2.03 ± 0.02 | 90.14 ± 0.45 | 1.86 ± 0.01 | 3.93 ± 0.07 | 3.24 ± 0.04 | 286.07 ± 4.88 | 30.71 ± 0.12 | 607.42 ± 20.93 | 646.67 ± 0.01 | 82.45 ± 2.12 | 52.14 ± 0.14 | 60.72 ± 0.02 |
| N33(out) | 2.02 ± 0 | 102.26 ± 1.29 | 2.58 ± 0.04 | 2.53 ± 0.02 | 5.18 ± 0.02 | 238.25 ± 21.78 | 33.38 ± 0.42 | 510.35 ± 22.6 | 526.11 ± 0.01 | 45.05 ± 0.02 | 48.91 ± 0.15 | 43.92 ± 0.03 |
| N34(out) | 3.84 ± 0.01 | 43.38 ± 0.12 | 0.08 ± 0 | 0.44 ± 0 | 2.45 ± 0.01 | 248.37 ± 6.18 | 19.65 ± 0 | 557.11 ± 5.02 | 544.07 ± 0.01 | 84.22 ± 0.01 | 40.7 ± 0.18 | 53.28 ± 0.01 |
| N35(out) | 2.14 ± 0.01 | 70.35 ± 0.23 | 3.84 ± 0.01 | 3.36 ± 0.02 | 7.68 ± 0.05 | 254.11 ± 16.91 | 28.38 ± 0.12 | 505.61 ± 7.53 | 495.74 ± 0.01 | 79.4 ± 0.24 | 53.37 ± 0.14 | 66.96 ± 0.05 |
| N36(out) | 1.12 ± 0.01 | 40.8 ± 0.4 | 1.83 ± 0.01 | 3.69 ± 0.03 | 3.35 ± 0.03 | 295.26 ± 1.63 | 15.14 ± 0.12 | 634.65 ± 15.9 | 587.78 ± 0.01 | 97.74 ± 0.12 | 41.33 ± 0.17 | 55.92 ± 0.03 |

Table S2. Validation parameters of the HPLC method for simultaneous determination of five phenolic compounds in BBL

| Parameter | Neochlorogenic acid | Chlorogenic acid | Rutin | Hyperoside | Isoquercitrin | Acceptance Criteria |
| --- | --- | --- | --- | --- | --- | --- |
| Linearity | | | | | | |
| Range (μg/mL) | 3.90 - 280.80 | 5.00 - 5000.00 | 5.00 - 240.00 | 3.00 - 150.00 | 5.00 - 360.00 |  |
| Slope | 9.9837 | 8.8682 | 17.999 | 26.037 | 23.298 |  |
| Intercept | -5.8752 | 136.58 | -2.5865 | -0.4796 | 3.8956 |  |
| *R^2^* | 1.000 | 0.999 | 0.999 | 1.000 | 1.000 | ≥ 0.995 |
| Sensitivity | | | | | | |
| LOD (μg/mL) | 1.09 | 0.49 | 0.13 | 0.28 | 0.59 | 3S/N |
| LOQ (μg/mL) | 3.60 | 1.62 | 0.41 | 0.94 | 1.97 | 10S/N |
| Precision (RSD%, n = 6) | | | | | | |
| System precision | 0.20% | 0.25% | 0.24% | 0.23% | 0.24% | ≤ 2% |
| Reproducibility | 1.17% | 1.16% | 0.83% | 1.40% | 1.22% | ≤ 3% |
| Accuracy (Recovery %) | | | | | | |
| Mean ± SD | 97.57 ± 1.25 | 101.91 ± 0.31 | 97.32 ± 0.69 | 97.93 ± 1.61 | 97.03 ± 0.72 | 95 - 105 |
| Stability (RSD%) | | | | | | |
| Solution stability (24 h) | 0.10% | 0.30% | 0.67% | 0.81% | 0.21% | ≤2% |

Table S3. Validation parameters for spectrophotometric determination of TPC and TFC in BBL

| Parameter | TPC | TFC | Acceptance Criteria |  |
| --- | --- | --- | --- | --- |
| Reference substance | Chlorogenic acid | Rutin |  |  |
| Linearity | | | | |
| Range (μg/mL) | 15.62 - 250.0 | 15.62 - 250.0 |  |  |
| Slope | 0.0029 | 0.0019 |  |  |
| Intercept | 0.0821 | 0.045 |  |  |
| *R^2^* | 0.996 | 1.000 | ≥ 0.995 |  |
| Sensitivity | | | | |
| LOD (μg/mL) | 0.95 | 0.90 | 3.3δ/S |  |
| LOQ (μg/mL) | 2.89 | 2.72 | 10δ/S |  |
| Precision (RSD%, n = 6) | | | | |
| System precision | 1.50 | 0.77 | ≤ 2% |  |
| Reproducibility | 1.80 | 1.40 | ≤ 3% |  |
| Accuracy (Recovery %) | | | | |
| Mean ± SD | 101.09 ± 1.20 | 98.11 ± 2.4 | 95 - 105 |  |
| Stability (RSD%) | | | | |
| Reaction solution stability (3 h) | 0.95 | 0.91 | ≤2 % |  |

Table S4. Correlation matrix of chemical compositions and bioactivities in BBL.

|  | Neochlorogenic acid | Chlorogenic acid | Rutin | Hyperin | Isoquercitrin | TPC | TFC | DPPH | ABTS | α-Glucosidase | Pancreatic lipase | Xanthine oxidase |
| --- | --- | --- | --- | --- | --- | --- | --- | --- | --- | --- | --- | --- |
| Neochlorogenic acid | 1.000 | 0.041 | 0.010 | -0.141 | 0.293** | 0.464*** | 0.051 | 0.450*** | 0.269** | 0.176 | 0.068 | -0.096 |
| Chlorogenic acid | 0.041 | 1.000 | 0.078 | 0.213* | 0.178 | 0.097 | 0.520*** | 0.191* | -0.005 | -0.139 | 0.072 | -0.173 |
| Rutin | 0.010 | 0.078 | 1.000 | -0.187 | 0.190* | 0.132 | 0.104 | 0.155 | 0.060 | 0.025 | -0.090 | 0.038 |
| Hyperin | -0.141 | 0.213* | -0.187 | 1.000 | -0.226* | -0.121 | 0.278** | -0.104 | -0.165 | -0.165 | -0.218* | 0.099 |
| Isoquercitrin | 0.293** | 0.178 | 0.190* | -0.226* | 1.000 | 0.417*** | 0.372*** | 0.488** | 0.337*** | 0.212* | 0.118 | -0.030 |
| TPC | 0.464*** | 0.097 | 0.132 | -0.121 | 0.417*** | 1.000 | 0.325*** | 0.874*** | 0.763*** | 0.588*** | -0.071 | -0.079 |
| TFC | 0.051 | 0.520*** | 0.104 | 0.278** | 0.372*** | 0.325*** | 1.000 | 0.272** | 0.303** | 0.037 | 0.146 | 0.055 |
| DPPH | 0.450*** | 0.191* | 0.155 | -0.104 | 0.488*** | 0.874*** | 0.272** | 1.000 | 0.711*** | 0.605*** | -0.229* | -0.203* |
| ABTS | 0.269** | -0.005 | 0.060 | -0.165 | 0.337*** | 0.763*** | 0.303** | 0.711*** | 1.000 | 0.503*** | -0.099 | -0.038 |
| α-Glucosidase | 0.176 | -0.139 | 0.025 | -0.165 | 0.212* | 0.588*** | 0.037 | 0.605*** | 0.503*** | 1.000 | -0.057 | -0.047 |
| Pancreatic lipase | 0.068 | 0.072 | -0.090 | -0.218* | 0.118 | -0.071 | 0.146 | -0.229* | -0.099 | -0.057 | 1.000 | 0.095 |
| Xanthine oxidase | -0.096 | -0.173 | 0.038 | 0.099 | -0.030 | -0.079 | 0.055 | -0.203* | -0.038 | -0.047 | 0.095 | 1.000 |

Note. ***, ** and * indicate statistically significant correlations at *P*<0.001, *P*<0.01 and *P*<0.05 levels, respectively.

Table S5. Comprehensive scores of BBL samples based on determined parameters

| Sample code | CompScore | Sample code | CompScore | Sample code | CompScore | Sample code | CompScore | Sample code | CompScore |
| --- | --- | --- | --- | --- | --- | --- | --- | --- | --- |
| R1(in) | 0.304 | R18(in) | 0.291 | S15(out) | 0.391 | N5(in) | 0.262 | N19(out) | 0.361 |
| R2(in) | 0.233 | R18(out) | 0.314 | S16(in) | 0.343 | N6(in) | 0.251 | N20(in) | 0.221 |
| R3(in) | 0.278 | R19(out) | 0.291 | S16(out) | 0.359 | N7(in) | 0.280 | N21(in) | 0.208 |
| R3(out) | 0.476 | S1(in) | 0.203 | S17(in) | 0.141 | N7(out) | 0.413 | N22(in) | 0.280 |
| R4(in) | 0.295 | S1(out) | 0.346 | S18(in) | 0.120 | N8(in) | 0.281 | N23(in) | 0.290 |
| R5(in) | 0.328 | S2(in) | 0.207 | S18(out) | 0.225 | N8(out) | 0.326 | N24(in) | 0.337 |
| R5(out) | 0.412 | S3(in) | 0.196 | S19(in) | 0.290 | N9(in) | 0.200 | N24(out) | 0.336 |
| R6(in) | 0.282 | S4(in) | 0.230 | S20(in) | 0.192 | N9(out) | 0.217 | N25(in) | 0.261 |
| R6(out) | 0.510 | S5(in) | 0.219 | S21(in) | 0.175 | N10(in) | 0.206 | N26(in) | 0.343 |
| R7(in) | 0.287 | S5(out) | 0.374 | S22(out) | 0.428 | N11(in) | 0.274 | N26(out) | 0.401 |
| R8(in) | 0.308 | S6(in) | 0.154 | S23(out) | 0.413 | N11(out) | 0.339 | N27(in) | 0.193 |
| R9(in) | 0.245 | S7(in) | 0.189 | S24(out) | 0.375 | N12(in) | 0.210 | N27(out) | 0.303 |
| R10(in) | 0.239 | S7(out) | 0.402 | S25(out) | 0.225 | N13(in) | 0.243 | N28(in) | 0.250 |
| R11(in) | 0.245 | S8(in) | 0.233 | S26(out) | 0.494 | N14(in) | 0.249 | N28(out) | 0.346 |
| R12(in) | 0.367 | S9(in) | 0.274 | S27(out) | 0.270 | N15(in) | 0.210 | N29(out) | 0.473 |
| R13(in) | 0.281 | S10(in) | 0.247 | S28(out) | 0.328 | N15(out) | 0.319 | N30(out) | 0.370 |
| R13(out) | 0.509 | S11(in) | 0.089 | S29(out) | 0.453 | N16(in) | 0.206 | N31(out) | 0.387 |
| R14(in) | 0.268 | S12(in) | 0.283 | N1(in) | 0.220 | N16(out) | 0.323 | N32(out) | 0.397 |
| R15(in) | 0.245 | S13(in) | 0.233 | N2(in) | 0.214 | N17(in) | 0.231 | N33(out) | 0.375 |
| R16(in) | 0.266 | S14(in) | 0.152 | N2(out) | 0.403 | N17(out) | 0.282 | N34(out) | 0.244 |
| R17(in) | 0.265 | S14(out) | 0.161 | N3(in) | 0.186 | N18(in) | 0.348 | N35(out) | 0.426 |
| R17(out) | 0.406 | S15(in) | 0.243 | N4(in) | 0.285 | N19(in) | 0.248 | N36(out) | 0.301 |


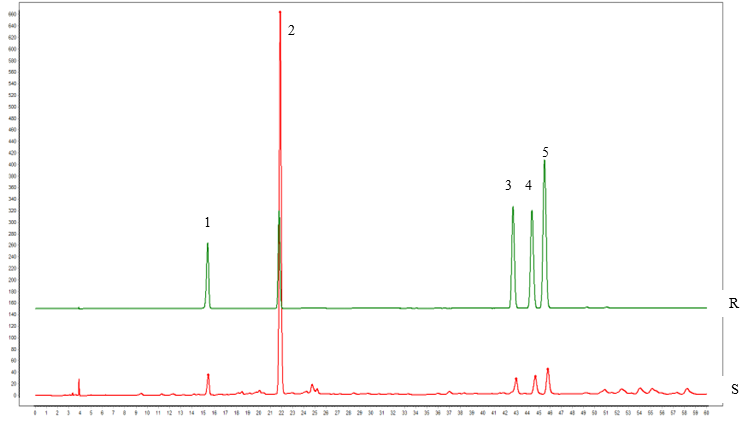


Fig. S1 Chromatographic separation (λ = 254 nm) of BBLextracts of reference substances and the sample. R denotes the mixed reference standards, S denotes the BBL sample.
